# Desmocollin-1 is associated with pro-metastatic phenotype of luminal A breast cancer cells and is modulated by parthenolide

**DOI:** 10.1186/s11658-023-00481-6

**Published:** 2023-08-24

**Authors:** Petr Lapcik, Petr Sulc, Lucia Janacova, Katerina Jilkova, David Potesil, Pavla Bouchalova, Petr Müller, Pavel Bouchal

**Affiliations:** 1 Department of Biochemistry, Faculty of Science, Masaryk University, Kamenice 5, 62500 Brno, Czech Republic; 2 Central European Institute of Technology, Masaryk University, Brno, Czech Republic; 3 Masaryk Memorial Cancer Institute, RECAMO, Brno, Czech Republic

**Keywords:** DIA, Proteomics, Pull-down, DSC1, Breast cancer, Metastasis

## Abstract

**Background:**

Desmocollin-1 (DSC1) is a desmosomal transmembrane glycoprotein that maintains cell-to-cell adhesion. DSC1 was previously associated with lymph node metastasis of luminal A breast tumors and was found to increase migration and invasion of MCF7 cells in vitro. Therefore, we focused on DSC1 role in cellular and molecular mechanisms in luminal A breast cancer and its possible therapeutic modulation.

**Methods:**

Western blotting was used to select potential inhibitor decreasing DSC1 protein level in MCF7 cell line. Using atomic force microscopy we evaluated effect of DSC1 overexpression and modulation on cell morphology. The LC–MS/MS analysis of total proteome on Orbitrap Lumos and RNA-Seq analysis of total transcriptome on Illumina NextSeq 500 were performed to study the molecular mechanisms associated with DSC1. Pull-down analysis with LC–MS/MS detection was carried out to uncover DSC1 protein interactome in MCF7 cells.

**Results:**

Analysis of DSC1 protein levels in response to selected inhibitors displays significant DSC1 downregulation (*p*-value ≤ 0.01) in MCF7 cells treated with NF-κB inhibitor parthenolide. Analysis of mechanic cell properties in response to DSC1 overexpression and parthenolide treatment using atomic force microscopy reveals that DSC1 overexpression reduces height of MCF7 cells and conversely, parthenolide decreases cell stiffness of MCF7 cells overexpressing DSC1. The LC–MS/MS total proteome analysis in data-independent acquisition mode shows a strong connection between DSC1 overexpression and increased levels of proteins LACRT and IGFBP5, increased expression of IGFBP5 is confirmed by RNA-Seq. Pathway analysis of proteomics data uncovers enrichment of proliferative MCM_BIOCARTA pathway including CDK2 and MCM2-7 after DSC1 overexpression. Parthenolide decreases expression of LACRT, IGFBP5 and MCM_BIOCARTA pathway specifically in DSC1 overexpressing cells. Pull-down assay identifies DSC1 interactions with cadherin family proteins including DSG2, CDH1, CDH3 and tyrosine kinase receptors HER2 and HER3; parthenolide modulates DSC1-HER3 interaction.

**Conclusions:**

Our systems biology data indicate that DSC1 is connected to mechanisms of cell cycle regulation in luminal A breast cancer cells, and can be effectively modulated by parthenolide.

**Graphical Abstract:**

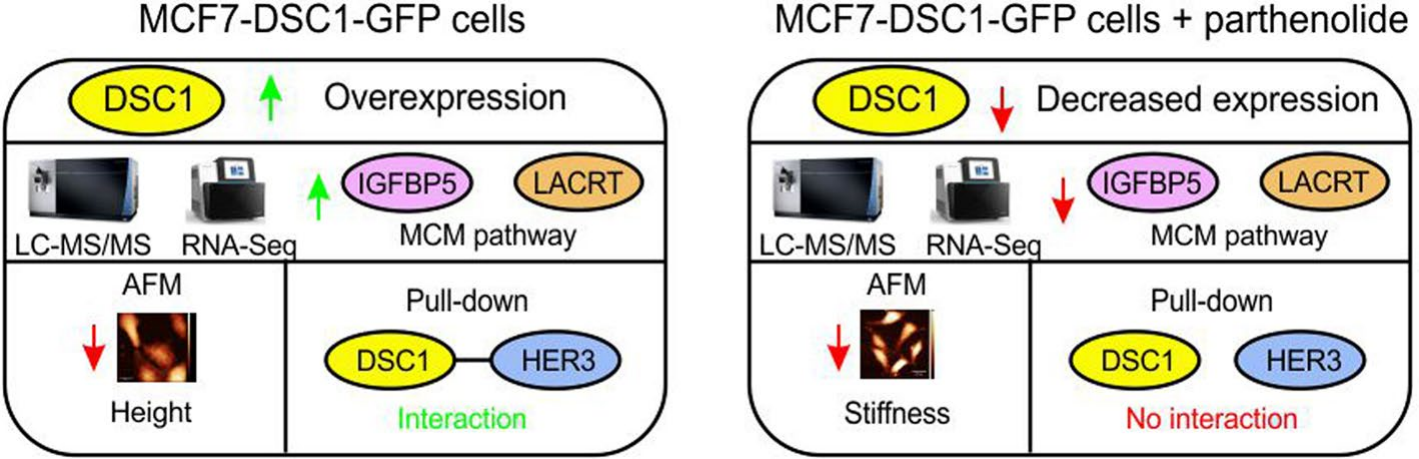

**Supplementary Information:**

The online version contains supplementary material available at 10.1186/s11658-023-00481-6.

## Introduction

Breast cancer represents the most prevalent and the most lethal cancer disease in the female population [1]. It is a heterogeneous disease that can be classified into intrinsic or molecular subtypes which differ in molecular profile, treatment strategy, response to treatment, and patient outcome [2]. These involve at least four molecular subtypes including luminal A, luminal B, HER2 + and triple negative [3, 4]. Generally, luminal A tumors that are the most prevalent and have the most favorable outcome express estrogen and progesterone receptors, do not express tyrosine-protein kinase erbB-2 (HER2/ERBB2) and show low levels of proliferation marker Ki-67 [5]. Although luminal B tumors also express hormonal receptors, these tumors can express HER2 and usually have higher proliferation potential associated with increased tumor aggressiveness [6]. On the other hand, HER2 + tumors lack hormonal receptors and overexpress HER2, whereas triple-negative tumors do not express any of these receptors. Both HER2 + and triple negative subtypes are associated with high grade and poor clinical prognosis [7]. For luminal A subtype, well established and effective treatment includes hormone therapy that targets estrogen and progesterone receptors and is based on tamoxifen or aromatase inhibitors [8, 9]. However, up to 16% of node-negative luminal A patients and up to a third of lymph node positive luminal A patients at the time of diagnosis develop distant metastasis [10, 11]. Moreover, about 20% to 30% of patients with estrogen receptor-positive tumors become resistant to the endocrine treatment [12]. Thus, development of more stratified approaches targeting the pro-metastatic mechanisms is of a high clinical need. For HER2 + subtype, trastuzumab treatment targeting HER2 receptor that is expressed on the surface of tumor cells, represents a prototype of a successful treatment that has reversed the prognosis of HER2 + patients to more favorable outcome.

In our previous study, we identified desmocollin-1 (DSC1) as a protein more abundant in more migrating population of MDA-MB-231 breast cancer cell line [13]. Subsequently, immunohistochemical analysis of 96 primary breast tumors revealed increased levels of DSC1 in lymph node positive luminal A tumors compared to their lymph node negative counterparts [13]. Increased levels of DSC1 were also observed in higher grade and HER2 + breast cancer subtypes and was associated with worse distant metastasis free survival in lymph node positive breast tumors [13]. Finally, DSC1 overexpression was associated with higher migration and invasiveness of MCF7 cells [13]. Generally, DSC1 is a transmembrane glycoprotein belonging to the cadherin family [14]. Cadherins participate in intercellular adhesion via desmosome formation and influence migration of cells [15], and potentially influence motility and invasion of tumor cells [16]. Based on these findings we hypothesize that DSC1 may play a role in metastasis of luminal A breast cancer and has a potential to serve as a therapeutic target. Here we aim at understanding the molecular role of DSC1 in breast cancer cells using proteomics [17] and RNA sequencing (RNA-Seq), analysis of its role in cell morphology, and at proposing the inhibitor that can modulate DSC1 protein levels and DSC1-related molecular mechanisms. Finally, we identify proteins interacting with DSC1 using pull-down assay and detect protein interactions targetable by DSC1 inhibition.

## Materials and methods

### Cultivation of cell lines

The MCF7 breast cancer cell line (ATCC, USA), stably transduced MCF7-DSC1-GFP line producing DSC1—Streptavidin-Binding Peptide—Green Fluorescent Protein (DSC1-SBP-GFP) fusion protein, and control MCF7-GFP line producing SBP-GFP fusion protein, both transduced lines prepared as described by Faktor et al. [13], were cultured in Dulbecco's Modified Eagle´s Medium (DMEM, Sigma-Aldrich, USA) supplemented with 10% fetal bovine serum (FBS, Sigma-Aldrich, USA) at 37 °C and 5% CO_2_ to approximately 80% confluency. Cells were washed with sterile 0.5% EDTA in 1 × phosphate buffered saline (1 × PBS; 0.137 M NaCl; 2.68 mM KCl; 1.47 mM KH_2_PO_4_; 6.45 mM Na_2_HPO_4_) during passaging, harvested using 0.125% trypsin solution (Sigma-Aldrich, USA) and resuspended in DMEM with 10% FBS. The growth medium was periodically evaluated for mycoplasma contamination.

For the inhibitor selection, 250,000 MCF7 cells for each condition were placed on three 6 cm plates in 4.5 ml complete DMEM media supplemented with 10% FBS. Cells were subsequently cultivated for 24 h prior the transient transfection.

For the atomic force microscopy (AFM) experiment, the 3 cm plates were coated with 1 ml of sterile poly-L-lysine solution in water for one hour and then the solution was aspirated off. Stably transduced MCF7-DSC1-GFP and control MFC7-GFP cells were counted using Bürker’s chamber and 80,000 cells were added to coated 3 cm plates. DMEM medium was filled to the total volume of 2 ml. Cells were further incubated with parthenolide at IC50 concentration or dimethyl sulfoxide (DMSO) for 24 h.

For the total proteome experiment, MCF7-DSC1-GFP cells and control MCF7-GFP cells were grown on 6-well plates as above in three biological replicates per condition. 300,000 cells were placed on the wells. After 24 h, cells were treated with parthenolide at IC50 concentration or DMSO and incubated for 24 h. Floating cells were stained using 0.4% trypan blue mixed 1:1 with cell suspension and counted using LUNA-II cell counter (Logos Biosystems, Inc.).

For the pull-down experiment, cell line MCF7-DSC1-GFP and control cell line MCF7-GFP were grown in three biological replicates, each replicate on three 15 cm plates to 70% confluency. Cells were further incubated with parthenolide at IC50 concentration or DMSO for 24 h.

For the RNA-Seq experiment, MCF7-DSC1-GFP and MCF7-GFP cells were cultured on 6 cm plates and were incubated with parthenolide at IC50 concentration or DMSO for 24 h. Cells were prepared in biological duplicates for each condition.

### Transient transfection of MCF7 cells

MCF7 cells were transiently transfected using pLenti6.3-rbs-DSC1-GWs-C-HA-IRES-GFP plasmid. Transfection solution was prepared in the volume of 500 μl/6 cm plate. The solution consisted of 4 μg plasmid DNA and 12 μl polyethylenimide, ratio 1:3 dissolved in DMEM medium without FBS in final volume of 500 μl. Solution was incubated for 15 min at RT. The solution was administered on plates with cells in the next step and incubated for 12 h at 37 °C and at 5% CO_2._ After 12 h DMEM medium was replaced with fresh DMEM. Transfection efficacy was checked using fluorescent microscopy.

### Selection of DSC1 inhibitor

Transiently transfected MCF7 cells were treated with inhibitors niclosamide (chemical purity 99.4%, Sigma-Aldrich), norcantharidine (chemical purity 99.5%, Sigma-Aldrich) and parthenolide (chemical purity 98%, Sigma-Aldrich) that were selected based on our previous study [11]. Each inhibitor was added to reach its IC50 concentration – 1.68 µM for niclosamide, 270.6 µM for norcantharidin and 12.8 µM for parthenolide. Control cells were treated with DMSO (Sigma-Aldrich). Cells were incubated for 24 h at 37 °C and 5% CO_2_. After incubation, significant number of cells lost its adherent state, hence growth media were centrifugated at 1000 × g for 5 min at RT. Adherent cells were washed 2 times with 1.5 ml 1 × PBS. Adherent and pelleted cells were harvested, pooled, and lysed in a buffer containing 250 mM Tris/HCl pH 6.8, 10% SDS, 30% glycerol, 5% β-mercaptoethanol and 0.02% bromophenol blue at 95 °C. Cell lysate was subsequently incubated at 95 °C for 10 min. Protein concentrations were determined using RC-DC protein assay kit (Bio-Rad, USA) according to manufacturer’s instructions.

### Western blot analysis

30 µg of a total protein was loaded on 10% separation and 5% concentration sodium dodecyl sulfate–polyacrylamide gel and electrophoretically separated as previously described [18]. Separated proteins were transferred onto a nitrocellulose blotting membrane BioTrace™ NT, pure Nitrocellulose, 0.22 μm (Pall Life Sciences, Mexico) in a blotting buffer (20% methanol, 0.19 M glycine, 24.8 mM Tris Base) at 100 V for 75 min. The efficiency of blotting was tested using Ponceau S solution (2.63 mM Ponceau S, 0.14 M sulfosalicylic acid, 0.18 M trichloroacetic acid). Membranes were blocked for 1 h in a 5% non-fat dried milk in 0.1% Tween + 1 × PBS. Next, rabbit polyclonal anti-DSC1 antibody (ab198904, Abcam, UK, dilution 1:1000) was used to detect DSC1. Primary antibody was diluted in a 5% non-fat dried milk in 0.1% Tween-20 + 1 × PBS. Mouse monoclonal anti-actin antibody clone AC-40 (A4700, Sigma-Aldrich, USA, dilution 1:1000) was used as a loading control. For detection of fusion proteins with SBP tag, anti-SBP antibody was used (MAB10764, Merck, USA, dilution 1:2000). Membranes were incubated overnight with primary antibodies at 4 °C and subsequently washed in 1 × PBS and 0.1% Tween + 1 × PBS both twice. Corresponding secondary antibodies conjugated with horseradish peroxidase were diluted in 5% non-fat dried milk in 0.1% Tween-20 + 1 × PBS to 1:1000 and incubated with membranes for 60 min at RT. Membranes were then washed again in PBS and 0.1% Tween + 1 × PBS both twice and incubated with freshly prepared enhanced chemiluminescence (ECL) solution composed from solution A (10 mM luminol, 0.5 mM EDTA, 405 μM coumaric acid, 200 mM Tris pH 9.4) and solution B (0.5 mM EDTA, 8 mM sodium perborate tetrahydrate, 50 mM sodium acetate pH 5) mixed in 1:1 ratio. ECL solution was removed after 5 min incubation and chemiluminiscence was detected on a Fusion Fx Spectra (Vilber Lourmat, France). Semiquantitative analysis of the signals was performed using QuantityOne 4.6 software (Bio-Rad, USA) with signal comparison of DSC1 to actin in Microsoft Excel using Student *t*-test, results were visualized in GraphPad Prism 8.4.3. As the best performing inhibitor, parthenolide was selected for the following experiments.

### Atomic force microscopy

After the growth medium replacement, cells were analyzed with AFM microscope Nano Wizard 3 (JPK Instruments, Germany) using AFM probe HYDRA-ALL (force mapping, contact mode, set point 100 nm; AppNano, USA) with tip in shape of tetrahedral pyramid. Data were evaluated in JPK Data Processing 5 software and Gwyddion 2.46 and afterwards illustrated by boxplots in GraphPad Prism 8.4.3 comparing the cell stiffness and cell height of the stably transduced MCF7-DSC1-GFP and MCF7-GFP treated with either parthenolide or DMSO. Statistical significance of the differences between these two cell lines in cell stiffness and cell height was calculated in GraphPad Prism 8.4.3 using a two-tailed Student *t*-test for normal data layout (*p* = 0.05).

### Sample preparation for total proteome analysis

During cell harvesting, DMEM media containing floating cells was removed and wells were washed twice with 1 × PBS, which was then added to the removed media and centrifuged at 1000 × *g*, 4 °C for 5 min. Supernatant was removed and pellet was resuspended in 1 × PBS. 1 × PBS was added to adherent cells in wells, which were scraped and pooled with cells from media. Cells were centrifuged at 1000 × *g*, 4 °C for 5 min. Lysis buffer (6 M guanidine hydrochloride, 0.1 M Na-phosphate, pH 6.6, 1% Triton X-100) was added to the wells to lyse the rest of unscraped cells to maintain the highest yield. This “conditioned” lysis buffer was used to lyse the pellets containing originally floating cells and cells scraped in 1 × PBS. Finally, samples were subsequently sonicated using HD 2200 (Bandelin, Germany) 30 × 0.1 s with power 50 W and after 30 s pause again 30 × 0.1 s with power 50 W. Cells were kept on ice during the sonication. Next, samples were incubated for 75 min at RT and centrifuged for 20 min at 4 °C and 14,000 × *g*. After centrifugation, supernatant was collected. Protein concentration was determined using RC-DC Protein Assay kit (Bio-Rad, USA).

### Pull-down assay

Growth medium was removed, cells were placed on ice and washed with ice cold 1 × PBS three times. Cells were than washed with 2 × concentrated Complete™, EDTA-free Protease Inhibitor Cocktail (Roche, Switzerland) in 1 × PBS. Another 2 × protease inhibitor cocktail was added, cells were scraped and centrifuged for 5 min at 1000 × *g*, 4 °C. Supernatant was removed and pelleted cells were stored at − 80 °C. Cell pellets placed on ice were resuspended in 1 ml HNN-lysis buffer (0.5% NP40, 0.2 M Na_3_VO_4_, 1 mM PMSF, 1 × Complete™, EDTA-free Protease Inhibitor Cocktail (Roche, Switzerland), 1.2 µM avidine). Cell suspension was incubated for 10 min on ice and centrifuged for 20 min at 13,000 ×* g*, 4 °C. During centrifugation, Micro BioSpin® 6 Columns (BioRad, USA) were equilibrated using 250 µl HNN-lysis buffer. 100 μl High Capacity Streptavidin Agarose Resin (Thermo Fisher Scientific, USA) was resuspended in 750 μl HNN-lysis buffer. Supernatants from samples were mixed with 200 μl streptavidin beads per sample. Samples were incubated on a rotation wheel for 15 min at 4 °C. Samples were subsequently transferred on equilibrated columns and washed twice with 1 ml HNN-lysis buffer and three-times with 1 ml HNN-buffer (50 mM HEPES pH 7.5, 150 mM NaCl, 50 mM NaF) without protease inhibitors and detergents. All washing steps were performed using gravity flow. Samples were finally eluted using 200 μl 2.5 mM biotin solution in HNN-buffer without protease inhibitors and detergents. Elution step was performed three times.

### Protein digestion and peptide desalting

Protein samples for total proteome and pull-down analysis were submitted to trypsin digestion using Filter-Aided Sample Preparation (FASP) method [19] and desalted as previously described [20]. Briefly, 100 µg of protein per total proteome sample or the whole pull-down eluates were transferred to the Microcon filter device, cut-off 30 kDa (Millipore, Germany) with 8 M urea in 0.1 M Tris/HCl, pH 8.5, reduced by tris(2-carboxyethyl)phosphine), alkylated using iodoacetamide, digested overnight by trypsin (Promega, USA) in the ratio 1:30, and resulting peptides were desalted on MicroSpin columns C18 (Nest Group, USA). Desalted peptides were dried in SpeedVac concentrator and stored at − 20 °C.

### LC–MS/MS measurement

The dried peptides were solubilized using 50 µl of 2.5% formic acid (FA) in 50% acetonitrile (ACN), then 100 µl of pure ACN was added and the samples were concentrated using SpeedVac concentrator (Thermo Fisher Scientific) to 20 µl. Finally, the concentrated samples were diluted into LC–MS vials to get peptide concentration of 0.8 µg/µl with addition of 1 µl 0.01% of polyethylene glycol (BioUltra, 20,000, Sigma-Aldrich) [21] in water, 1 µl of stock iRT peptides standard (Biognosys), 2 µl of 5% FA and filled into 10 µl by MilliQ water (Merck). Two µg of peptides mixture was injected for each sample.

Liquid chromatography-tandem mass spectrometry (LC–MS/MS) analyses were done using RSLCnano system online connected to Orbitrap Fusion™ Lumos™ tribrid mass spectrometer (Thermo Fisher Scientific, Waltham, MA, USA). Prior to LC separation, tryptic digests were online concentrated and desalted using trapping column (100 μm × 30 mm) filled with 3.5-μm X-Bridge BEH 130 C18 sorbent (Waters, Milford, MA, USA). After washing of trapping column with 0.1% FA, the peptides were eluted from the trapping column onto analytical Acclaim Pepmap100 C18 column (3 µm particles, 75 μm × 500 mm; Thermo Fisher Scientific, Waltham, MA, USA) by the following gradient program (mobile phase A: 0.1% FA in water; mobile phase B: 0.1% FA in 80% ACN; flow 300 nl/min): the gradient elution started at 5% of mobile phase B and began increase in the 5th min to 37% during the 109 min, then reached to 80% of mobile phase B in the next 6 min and remained at this state for the last 10 min. Equilibration of the trapping column and the analytical column was done prior to sample injection to sample loop. The analytical column outlet was directly connected to the Digital PicoView 550 (New Objective) ion source. Active Background Ion Reduction Device (ESI Source Solutions) was installed. MS data were acquired in a data-independent acquisition (DIA) mode.

Orbitrap analyzer and quadrupole mass filter were employed for survey scan detection (350–1650 m/z). The MS scan resolution was 120,000 (at 200 m/z) with a target value of 2 × 10^5^ ions and maximum injection time of 100 ms. After the MS scan, defined m/z segments were isolated by quadrupole mass filter and higher-energy collisional dissociation (HCD) fragmentation was done with a target value of 5 × 10^5^ ions. MS/MS spectra after HCD fragmentation (default charge state is 2 and 28% collision energy) were recorded in Orbitrap with a resolution of 30,000 (at 200 m/z) in scan range of 200–1800 m/z. The maximum injection time for MS/MS was 50 ms.

### Mass spectrometry data processing

DIA data were analyzed in Spectronaut software (Biognosys) version 13.6 for total proteome experiment and 13.9 for pull-down experiment, both in directDIA mode. UniProt/SwissProt database version 2019_07 downloaded on 2019-09-16 limited to human entries containing 20,431 sequences was used for database searches. Carbamidomethylation (C) was used as fixed modification, oxidation (M) and acetylation (protein N-term) were used as variable modifications. *q*-value at both precursor and protein levels were set to 0.01. For the total proteome experiment, data based on *q*-value 0.25 percentile (identified in 3 of 12 total runs) were involved in the final dataset. For the pull-down analysis, data based on *q*-value 0.33 percentile (identified in 3 of 9 total runs) were involved in the final dataset. Analysis of differential protein abundance was performed using *t*-test implemented in Spectronaut with false discovery rate correction. Default settings were used for other parameters.

### Sample preparation for transcriptomics analysis

The cells were washed two times with cold 1 × PBS on ice. The cells were harvested by adding 500 µl of 1 × PBS and scraping by a cell scraper, transferred to an Eppendorf tube, and stored on ice. After centrifuging (1000 × *g*, 4 °C, 5 min), the supernatant was aspirated and 360 µl of TRI reagent was added to the pelleted cells. Total RNA was isolated according to TRI reagent protocol (T9424, Sigma-Aldrich) and its concentration was determined using Qubit RNA BR assay kit (Q10210, Thermo Fisher Scientific) and RNA integrity and quality (cut-off > 8.0) using Qubit RNA IQ Assay Kit (Q33221, Thermo Fisher Scientific). 240 ng of total RNA at 20 ng/µl was used in RNA-Seq analysis. Samples were stored at − 80 °C.

### RNA-Seq analysis

The TruSeq Stranded Total RNA LT Sample Prep Kit (Illumina) was used to convert 0.5 mg of total RNA into a library of template molecules. Library was validated using Bioanalyzer (DNA 1000 Kit, Agilent) and quantified according to manufacturer instructions by qPCR (KAPA Library Quantification Kit Illumina platforms, Kapa Biosystems) using Quant studio (QuantStudio 5, Thermo Fisher Scientific). Samples were sequenced using NextSeq 500 (Illumina).

### RNA-Seq data processing

For RNA-seq, the raw reads were filtered to remove the adaptors and low-quality bases using Trimmomatic (v0.36) with Truseq2 s well as any reads that were shorter than 65 bases. Filtered reads were aligned to the human genome (Homo_sapiens.GRCh38.dna.primary_assembly) using STAR (v2.5.2b) in end-to-end mode to scan splice junctions. Then the counts in exon genomic features were calculated subread (v1.5.2). Differential expression analysis was performed in R 3.5.3 under the Deseq2 package version 1.22.2.

### Gene set enrichment analysis

Gene set enrichment analysis (GSEA) in GSEA Java desktop application [22] version 4.3.2 was conducted using the list of all quantified proteins from total proteome analysis pre-ranked according to the negative log2 of the *q*-value and the sign of the log2 FC to identify enriched pathways, with a priori defined pathways from BioCarta database. Minimal size of a gene set was adjusted to 10, otherwise default settings were used.

GSEA analysis of pull-down data was performed with minimal gene set size set to 2 and with use of Gene Ontology Biological Process (GOBP) database. False positive and false negative interacting partners (i) with log2 FC > 0 in pull-down and simultaneously log2 FC > 0 and *q*-value < 0.05 in total proteome analysis, or (ii) with log2 FC < 0 in pull-down and simultaneously log2 FC < 0 and *q*-value < 0.05 in total proteome analysis, were excluded from the pull-down GSEA analysis.

GSEA analysis of the RNA-Seq data was performed with the same settings as in the total proteome experiment for all quantified protein-coding transcripts with use of the BioCarta database.

## Results

### NF-κB inhibitor parthenolide modulates DSC1 expression

**Fig. 1:**
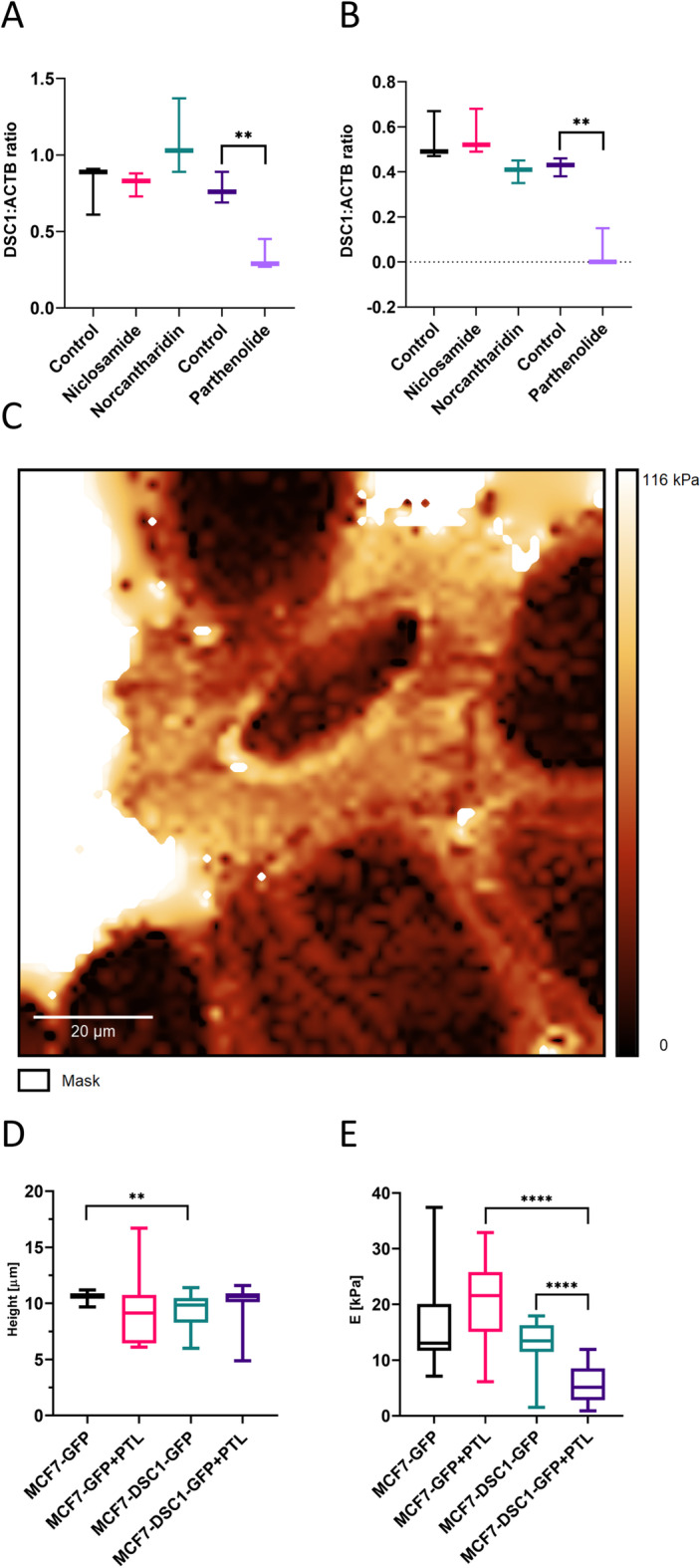
Statistical evaluation of semiquantitative analysis of inhibitor effects on DSC1 **A** preprotein **B** active protein levels. AFM was used to measure stiffness and height of MCF7-DSC1-GFP and control MCF7-GFP cell line (**C**). Comparison of **D** height and **E** stiffness of cells MCF7-DSC1-GFP and MCF7-GFP treated with parthenolide (PTL) and control cells. **p-value < 0.01, ****p-value < 0.0001

In the present study, we tested nuclear factor kappa-light-chain-enhancer of activated B cells (NF-κB) inhibitor parthenolide, cyclin-dependent kinase 2 (Cdk2) inhibitor norcantharidin and NF-κB inhibitor niclosamide to identify inhibitor decreasing protein levels of DSC1. Inhibitor experiments were performed in MCF7 cell line transiently transfected with a vector carrying *DSC1* gene and control cells. Significant decrease of both longer (Fig. 1A, *p*-value < 0.01) and shorter isoform (Fig. 1B, *p*-value < 0.01) of DSC1 protein were found in cells treated with parthenolide compared to the control, other inhibitors did not exhibit any effect (Fig. 1A, B, Additional file 1: Fig. S1 and Table S1). These results suggest that parthenolide has a significant downregulating effect on DSC1 protein levels and that DSC1 is sensitive to potential anti-metastatic inhibitors in luminal breast cancer cells.

### Parthenolide decreases stiffness of MCF7 cells specifically overexpressing DSC1

Based on the above findings, we investigated whether parthenolide is effective in functional experiments on both cellular and molecular level. Cell stiffness and height of the stably transduced MCF7-DSC1-GFP cell line were compared to MCF7-GFP cells using AFM (Fig. 1C). Both cell lines were treated with either parthenolide or DMSO as a control. Height of MCF7-DSC1-GFP cells was significantly (*p*-value = 5.66E−3) lower compared to MCF7-GFP cells (Fig. 1D). Parthenolide specifically and significantly (*p*-value = 6.71E−6) decreased stiffness of cells overexpressing DSC1 (Fig. 1E). These results indicate that DSC1 overexpression and parthenolide treatment affect MCF7 cell line morphology.

### DSC1 overexpression is associated with upregulation of proliferative pathways in MCF7 cells that are targetable by parthenolide

To characterize molecular mechanisms associated with DSC1 overexpression and parthenolide-induced DSC1 inhibition in MCF7 cell line, total proteome and transcriptome changes in MCF7-DSC1-GFP cells overexpressing DSC1 and control MCF7-GFP cells with parthenolide or DMSO treatment were evaluated. The adherent cells were harvested together with the floating cells which represent mixture of living and dead cells with 28.01 ± 4.26% and 37.99 ± 4.12% viability in MCF7-DSC1-GFP and MCF7-GFP cells treated with parthenolide, respectively, illustrating the activity of the inhibitor. Proteins from the cell lysates were identified and quantified using LC–MS/MS analysis in DIA mode and transcripts were identified and quantified using RNA-Seq approach.

3505 protein groups (FDR < 0.01, Additional file 2, for representative iRT peptide chromatograms please see Additional file 1: Fig. S2) and 17,157 protein-coding transcripts (Additional file 3) were quantified. DSC1 overexpression confirmed by increased DSC1 protein (*q*-value = 5.53E−49, log2 FC = 4.07) and transcript (*q*-value = 6.2E−129, log2 FC = 14.02) levels led to statistically significant (*q*-value < 0.05) up-regulation (protein log2 FC > 0.58, transcript log2 FC > 1) and down-regulation (protein log2 FC < − 0.58, transcript log2 FC < − 1) of 151 and 129 proteins, respectively (Additional file 2), and 276 and 268 transcripts, respectively (Additional file 3) (MCF7-DSC1-GFP vs. MCF7-GFP comparison). The most up-regulated proteins include extracellular glycoprotein lacritin (LACRT) and insulin-like growth factor-binding protein 5 (IGFBP5) that plays a role in cell proliferation, apoptosis, and survival (Additional file 1: Table S2). Increased protein levels were observed also for groups of proteins involved in cell proliferation (USP8, KIF23, RACGAP1, NUMA1, STMN1 and IQGA3) and cell adhesion (FREM2, TMOD3, NRCAM, BCAM) (Additional file 1: Table S2). Total of 14 genes displayed increased expression on both protein and transcript level in DSC1 overexpressing cells (Table 1) with IGFBP5 as the most stimulated gene at the protein level.

**Table 1:** Genes upregulated after DSC1 overexpression both on protein (*q*-value < 0.05, log2 FC > 0.58) and mRNA level (*q*-value < 0.05, log2 FC > 1)

Gene name	UniProt ID	Protein description	MCF7-DSC1-GFP vs. MCF7-GFP				MCF7-DSC1-GFP + PTL vs. MCF7-DSC1-GFP				MCF7-GFP + PTL vs. MCF7-GFP				MCF7-DSC1-GFP + PTL vs. MCF7-GFP + PTL			
			Proteomics		Transcriptomics		Proteomics		Transcriptomics		Proteomics		Transcriptomics		Proteomics		Transcriptomics	
			log2 FC	*q*-value	log2 FC	*q*-value	log2 FC	*q*-value	log2 FC	*q*-value	log2 FC	*q*-value	log2 FC	*q*-value	log2 FC	*q*-value	log2 FC	*q*-value
DSC1	Q08554	Desmocollin-1	4.07	4.98E−49	14.02	6.21E−129	− 0.52	3.31E−28	0.06	0.869	− 1.19	0.027	− 1.38	0.344	4.74	2.07E−40	15.45	1.53E−70
IGFBP5	P24593	Insulin-like growth factor-binding protein 5	2.87	2.92E−04	1.84	2.76E−40	− 2.50	0.014	− 1.03	8.17E−13	− 1.01	0.042	− 2.27	1.63E−60	1.39	0.089	3.08	4.08E−111
MYO5C	Q9NQX4	Unconventional myosin-Vc	2.59	1.10E−03	2.14	2.76E−68	− 0.36	0.010	0.12	0.546	− 0.58	0.137	0.02	0.938	2.81	3.40E−03	2.23	2.34E−74
SPPL2A	Q8TCT8	Signal peptide peptidase-like 2A	2.55	3.83E−04	2.08	3.40E−67	− 0.21	0.141	0.33	0.021	− 0.46	0.073	0.27	0.076	2.81	1.83E−03	2.14	6.81E−71
SLC27A2	O14975	Very long-chain acyl-CoA synthetase	2.37	1.17E−08	1.74	3.52E−14	− 0.55	7.61E−05	− 0.22	0.501	− 0.88	1.36E−03	− 1.24	1.16E−06	2.70	2.03E−07	2.75	7.14E−32
USP8	P40818	Ubiquitin carboxyl-terminal hydrolase 8	2.21	7.99E−07	1.88	6.02E−48	0.24	5.70E−05	0.46	1.45E−03	0.45	0.016	0.38	0.014	2.00	3.92E−11	1.97	2.86E−53
FREM2	Q5SZK8	FRAS1-related extracellular matrix protein 2	1.44	2.20E−14	1.12	1.07E−22	2.28	1.14E−06	− 1.44	7.03E−38	3.59	4.46E−03	− 1.67	9.36E−51	0.14	0.131	1.35	1.61E−32
RHOB	P62745	Rho-related GTP-binding protein RhoB	1.24	1.17E−07	1.06	7.28E−10	− 0.55	1.26E−06	− 0.11	0.695	0.12	0.162	0.46	0.013	0.57	2.52E−04	0.48	0.017
TMOD3	Q9NYL9	Tropomodulin-3	1.06	8.46E−16	1.70	1.90E−45	0.16	1.42E−03	0.23	0.131	0.29	2.09E−04	0.00	0.992	0.93	2.21E−14	1.93	1.40E−58
TOM1L1	O75674	TOM1-like protein 1	1.03	1.01E−06	1.18	1.16E−21	0.11	7.91E−03	− 0.07	0.728	0.18	0.039	− 0.17	0.302	0.95	1.38E−04	1.28	3.87E−25
FKBP10	Q96AY3	Peptidyl-prolyl cis–trans isomerase FKBP10	0.95	0.013	1.24	7.16E−14	− 0.46	0.021	− 0.73	2.97E−05	− 0.67	5.26E−04	− 1.44	7.32E−17	1.17	0.017	1.95	1.65E−30
CKMT1A	P12532	Creatine kinase U-type, mitochondrial	0.94	7.41E−07	1.32	1.47E−06	− 0.41	2.53E−03	− 0.29	0.412	− 0.67	1.37E−06	0.01	0.984	1.20	8.28E−09	1.01	4.75E−04
LXN	Q9BS40	Latexin	0.86	2.51E−12	2.57	1.01E−65	− 0.33	1.39E−03	1.18	1.60E−14	0.20	0.020	1.21	7.20E−15	0.34	8.17E−06	2.53	6.06E−66
MYO5A	Q9Y4I1	Unconventional myosin-Va	0.85	7.98E−05	1.72	5.19E−33	− 0.06	0.125	0.75	6.53E−07	− 0.38	0.023	0.49	2.30E−03	1.18	2.42E−04	1.98	2.39E−44
LMCD1	Q9NZU5	LIM and cysteine-rich domains protein 1	0.83	1.40E−03	1.45	1.44E−13	0.26	8.84E−03	1.54	6.55E−18	0.59	1.57E−03	1.59	5.95E−17	0.51	0.011	1.39	1.57E−14

*PTL* parthenolide

On the other hand, parthenolide treatment of DSC1 overexpressing cells (MCF7-DSC1-GFP + PTL vs. MCF7-DSC1-GFP comparison) was associated with significant down-regulation of 274 proteins and 655 transcripts. On the protein level, the most deregulated were LACRT and IGFBP5 (Additional file 1: Table S2). From the genes with increased expression both on protein and transcript level after DSC1 overexpression, only IGFBP5 displayed decreased protein and transcript levels in DSC1 overexpressing cells treated with parthenolide (Table 1), however DSC1 protein downregulation by parthenolide was observed also in control cells (MCF7-GFP + PTL vs. MCF7-GFP comparison). Parthenolide alters DSC1-induced up-regulation of LACRT and IGFBP5 proteins in MCF7-DSC1-GFP cells compared to control MCF7-GFP cells, both treated with parthenolide (MCF7-DSC1-GFP + PTL vs. MCF7-GFP + PTL comparison). These results suggest LACRT and IGFBP5 as the most co-expressed genes with DSC1 in breast cancer cells that can be modulated with parthenolide treatment.

**Fig. 2:**
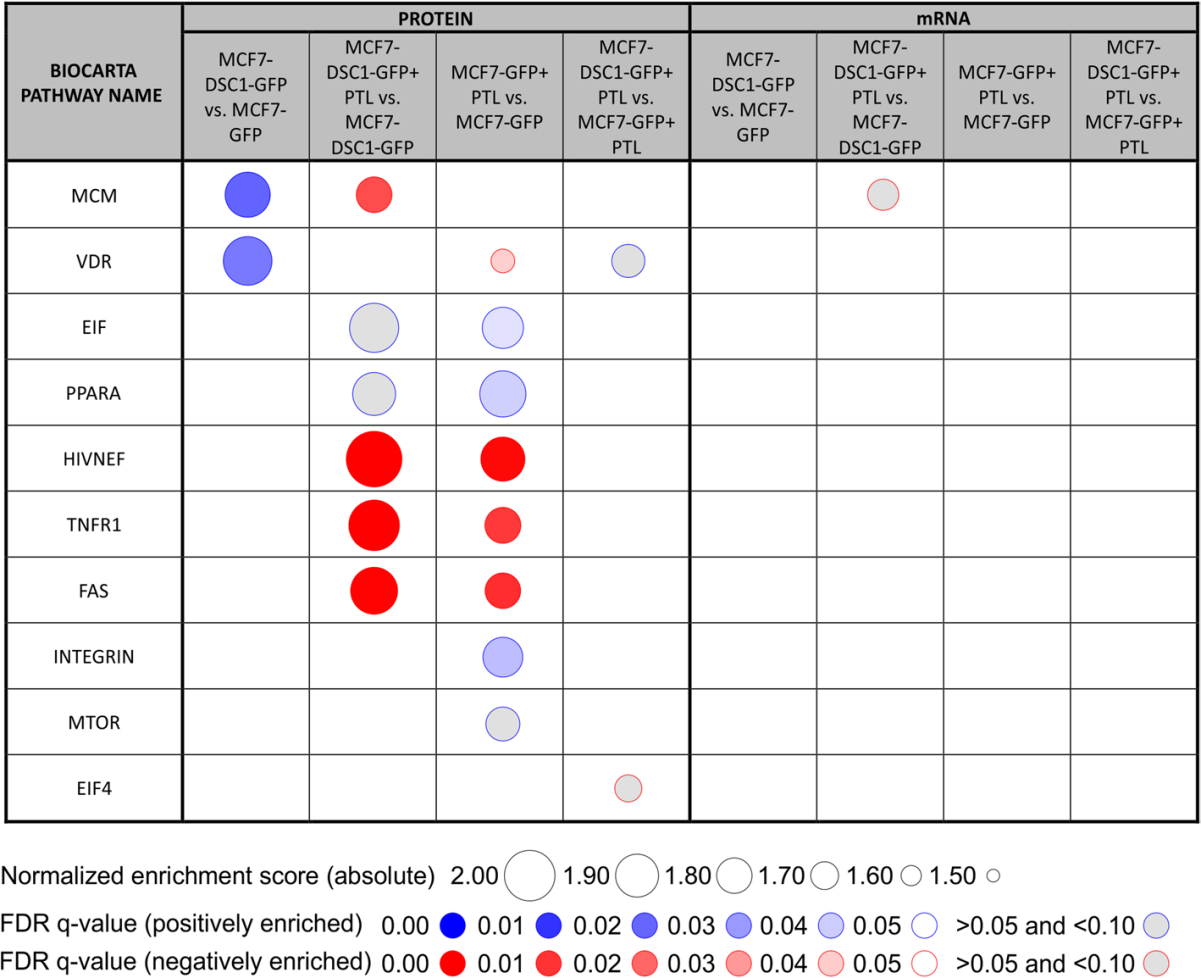
Gene set enrichment analysis of proteomics and transcriptomics profile comparisons of MCF7-DSC1-GFP and MCF7-GFP cells treated and untreated with parthenolide. *PTL* parthenolide

GSEA analysis was performed to define biological pathways associated with DSC1 overexpression and parthenolide-induced DSC1 inhibition on protein and transcript levels. DSC1 overexpression in MCF7 cells was associated with statistically significant (FDR *q*-value < 0.05) positive enrichment of 2 protein BIOCARTA pathways (Fig. 2), namely MCM and VDR pathways, from which MCM pathway consists of Cdk2 and helicases minichromosome maintenance (MCM) 2–7, and VDR pathway contains proteins involved in chromatin remodeling (Additional file 4). Parthenolide treatment of DSC1 overexpressing cells resulted in negative enrichment of 4 pathways, including MCM pathway. However, the parthenolide-induced negative enrichment of MCM pathway was specific for DSC1 overexpressing cells as the parthenolide did not induce negative enrichment of MCM pathway in the control cell line (Fig. 2). The negative enrichment of MCM pathway after parthenolide treatment specifically in DSC1 overexpressing cell line was observed also on the transcriptome level, although with lesser statistical significance (FDR *q*-value = 0.061) (Fig. 2, Additional file 4). These results indicate DSC1 overexpression in MCF7 cells to be associated with increased expression of genes involved in DNA replication and cell cycle progression.

In conclusion, DSC1 overexpression in MCF7 cells is related to increased expression of LACRT and IGFBP5 gene and proliferative MCM pathway. Parthenolide decreased LACRT and IGFBP5 protein and IGFBP5 transcript levels and suppressed MCM pathway specifically in DSC1 overexpressing MCF7 cells.

### Parthenolide modulates interactions of DSC1 with cell adhesion molecules and HER3

**Fig. 3:**
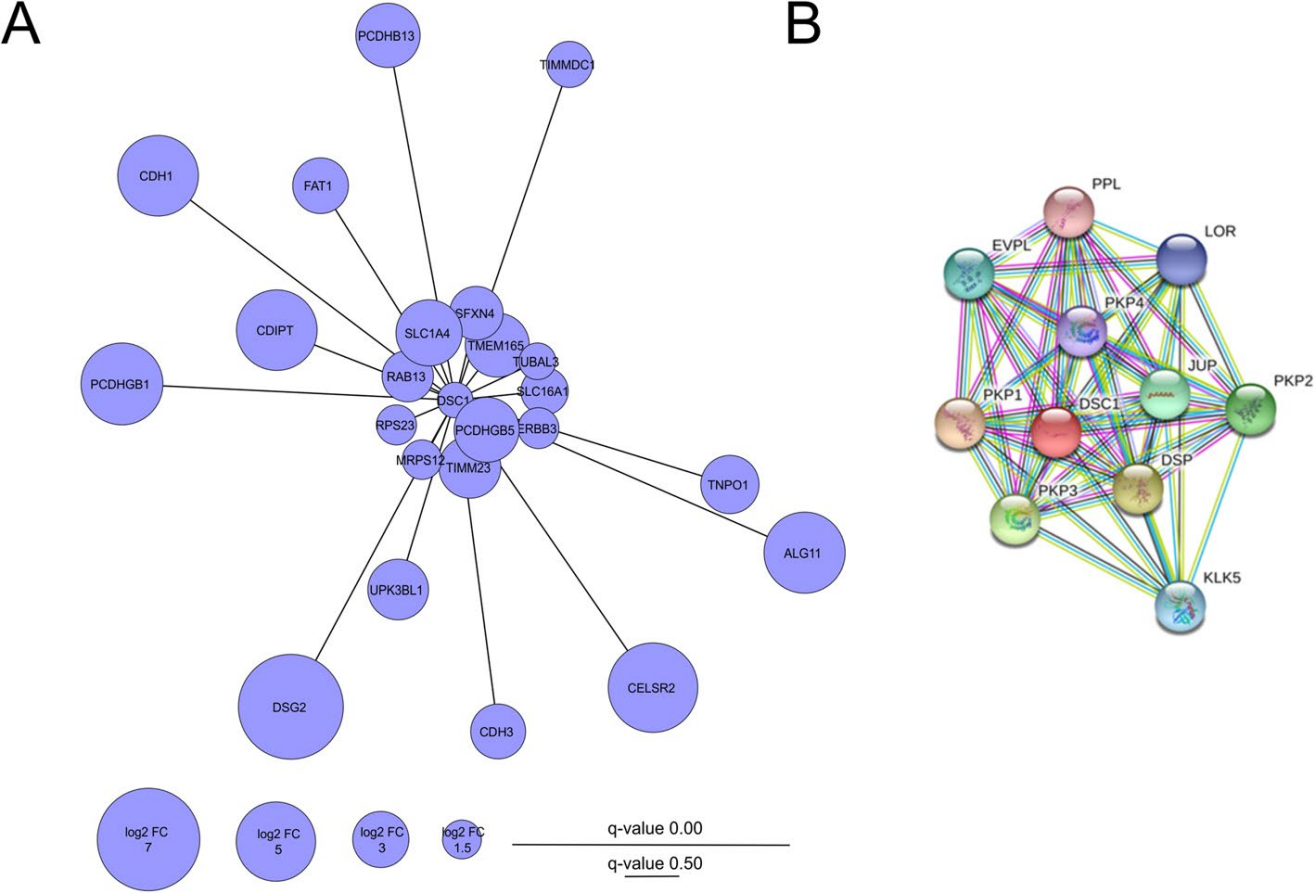
**A** 23 potential interaction partners of DSC1 modulated by parthenolide. **B** Known interaction partners of DSC1 in the STRING database

We performed a pull-down experiment to identify new potential DSC1 interacting partners binding to its N-terminus, as the DSC1-SBP-GFP fusion protein produced in MCF7-DSC1-GFP cells contains SBP tag attached to the C-terminus of DSC1, and to evaluate the ability of parthenolide to modulate these interactions. Presence of SBP tag in MCF7-DSC1-GFP cell line and control MCF7-GFP line was confirmed using western blot with immunodetection (Additional file 1: Fig. S3). Analysis of interacting partners in vitro was performed using pull-down assay with subsequent identification by LC–MS/MS in DIA mode. Total of 706 proteins were identified (Additional file 5). From these, 250 proteins are potential interacting partners of DSC1 (*q*-value < 0.05 and log2 FC > 1 in pull-down comparison MCF7-DSC1-GFP vs. MCF7-GFP), from which 54 were significantly downregulated by parthenolide (*q*-value < 0.05 and log2 FC < − 1 in pull-down comparison MCF7-DSC1-GFP + PTL vs. MCF7-DSC1-GFP). Pull-down results were compared with results of total proteome analysis to prevent from false positive interactions that could be caused by increased protein abundancy in pull-down due to gene co-expression with DSC1. Out of 250 potential interacting partners, 184 proteins (Additional file 5) were not found co-expressed with DSC1 in total proteome analysis (log2 FC < 0 or log2 FC > 0 and *q*-value > 0.05 or not identified in MCF7-DSC1-GFP vs. MCF7-GFP comparison in total proteome analysis). 23 of these not co-expressed potential interactors (Tab. 2, Fig. 3A) were modulated by parthenolide (*q*-value < 0.05 and log2 FC <  1 in pull-down comparison MCF7-DSC1-GFP + PTL vs. MCF7-DSC1-GFP). These proteins include desmoglein 2 (DSG2) that is essential for desmosome formation and is a known interactor of DSC1, which validates the results of this experiment. Moreover, cadherins 1 (CDH1) and cadherin 3 (CDH3), four protocadherins and cadherin receptor CELR2 that play role in cell adhesion were shown to interact with DSC1 as well. Parthenolide also inhibits interaction of DSC1 with receptor tyrosine-protein kinase erbB-3 (HER3/ERBB3) that is involved in cell proliferation.

**Table 2:** 23 potential interacting partners of DSC1 identified using pull-down assay that can be modulated with parthenolide

Protein name	Protein description	UniProt ID	Gene name	Pull-down MCF7-DSC1-GFP vs. MCF7-GFP		Pull-down MCF7-DSC1-GFP + PTL vs. MCF7-DSC1-GFP		Total proteome MCF7-DSC1-GFP vs. MCF7-GFP		Total proteome MCF7-DSC1-GFP + PTL vs. MCF7-DSC1-GFP	
				log2 FC	*q*-value	log2 FC	*q*-value	log2 FC	*q*-value	log2 FC	*q*-value
DSG2	Desmoglein-2	Q14126	DSG2	7.21	6.82E−53	− 2.01	1.70E−52	− 0.45	0.106	0.01	2.64E−04
CELR2	Cadherin EGF LAG seven-pass G-type receptor 2	Q9HCU4	CELSR2	5.88	7.04E−53	− 2.03	1.29E−45	NA	NA	NA	NA
PCDGD	Protocadherin gamma-B1	Q9Y5G3	PCDHGB1	5.22	8.02E−06	− 1.32	1.60E−05	NA	NA	NA	NA
ALG11	GDP-Man:Man(3)GlcNAc(2)-PP-Dol alpha-1,2-mannosyltransferase	Q2TAA5	ALG11	5.15	4.75E−10	− 2.24	2.35E−10	NA	NA	NA	NA
CADH1	Cadherin-1	P12830	CDH1	5.11	1.20E−14	− 1.39	1.28E−13	− 0.02	1.79E−03	− 0.09	0.224
CDIPT	CDP-diacylglycerol–inositol 3-phosphatidyltransferase	O14735	CDIPT	5.11	2.56E−09	− 2.30	1.45E−06	− 0.35	2.00E−03	− 0.01	0.122
SATT	Neutral amino acid transporter A	P43007	SLC1A4	3.86	5.27E−04	− 2.41	4.09E−03	NA	NA	NA	NA
PCDGH	Protocadherin gamma-B5	Q9Y5G0	PCDHGB5	3.77	6.88E−04	− 1.10	5.46E−04	NA	NA	NA	NA
PCDBD	Protocadherin beta-13	Q9Y5F0	PCDHB13	3.70	1.82E−06	− 2.29	1.71E−03	NA	NA	NA	NA
TM165	Transmembrane protein 165	Q9HC07	TMEM165	3.68	3.12E−03	− 1.33	4.51E−03	− 0.41	9.12E−06	− 0.08	0.234
TIM23	Mitochondrial import inner membrane translocase subunit Tim23	O14925	TIMM23	3.50	1.02E−03	− 1.25	0.032	0.10	0.131	− 0.03	0.244
UPK3L	Uroplakin-3b-like protein 1	B0FP48	UPK3BL1	3.39	1.13E−10	− 1.08	5.46E−04	NA	NA	NA	NA
TNPO1	Transportin-1	Q92973	TNPO1	3.18	8.96E−06	− 1.16	4.33E−03	0.02	0.139	0.17	9.29E−03
FAT1	Protocadherin Fat 1	Q14517	FAT1	2.96	1.58E−14	− 1.98	4.15E−12	NA	NA	NA	NA
CADH3	Cadherin-3	P22223	CDH3	2.89	5.37E−06	− 2.21	2.58E−03	NA	NA	NA	NA
SFXN4	Sideroflexin-4	Q6P4A7	SFXN4	2.78	2.14E−03	− 2.96	3.17E−03	NA	NA	NA	NA
RAB13	Ras-related protein Rab-13	P51153	RAB13	2.58	3.25E−03	− 1.94	0.026	0.04	0.175	− 0.16	0.154
MOT1	Monocarboxylate transporter 1	P53985	SLC16A1	2.24	6.75E−03	− 1.39	7.07E−03	− 0.71	2.77E−03	0.22	0.092
TIDC1	Complex I assembly factor TIMMDC1, mitochondrial	Q9NPL8	TIMMDC1	2.13	3.06E−04	− 1.37	4.51E−03	− 0.43	8.16E−03	− 0.06	0.197
ERBB3	Receptor tyrosine-protein kinase erbB-3	P21860	ERBB3	1.68	0.031	− 2.12	5.94E−03	NA	NA	NA	NA
RS23	40S ribosomal protein S23	P62266	RPS23	1.61	0.019	− 1.53	0.020	− 0.09	0.107	− 0.19	0.253
RT12	28S ribosomal protein S12, mitochondrial	O15235	MRPS12	1.56	8.30E−03	− 1.53	0.011	NA	NA	NA	NA
TBAL3	Tubulin alpha chain-like 3	A6NHL2	TUBAL3	1.32	0.047	− 1.82	0.045	− 0.23	0.166	− 0.34	0.056

*PTL* parthenolide

**Fig. 4:**
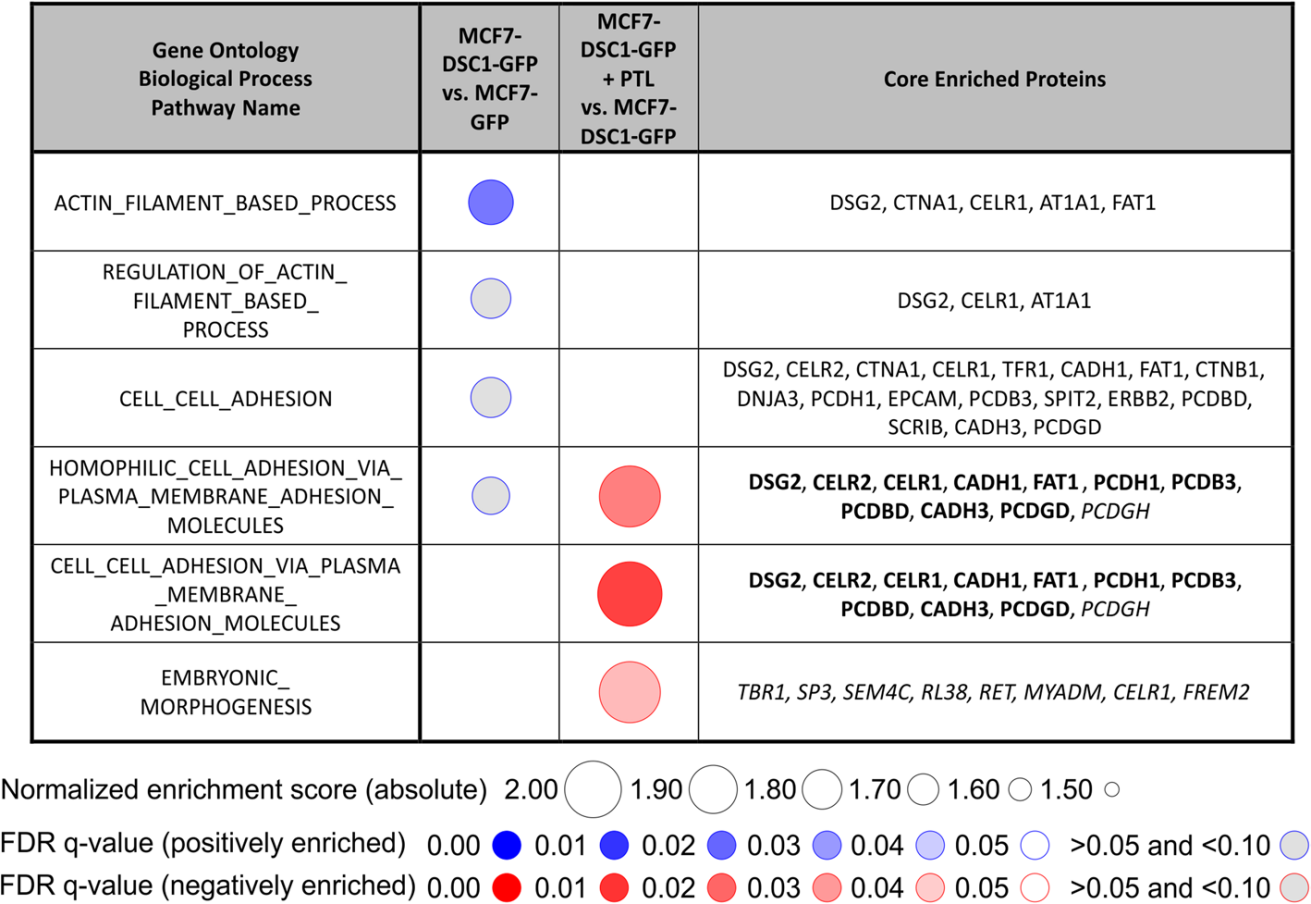
Gene set enrichment analysis of proteins that are not co-expressed with DSC1 and were identified in pull-down from MCF7-DSC1-GFP vs. MCF7-GFP cells treated and untreated with parthenolide. Proteins in italics were enriched in comparison MCF7-DSC1-GFP + PTL vs. MCF7-DSC1-GFP only. Proteins in bold were enriched in both comparisons. *PTL* parthenolide

GSEA of the pull-down identified proteins (Fig. 4) highlights participation of DSC1 potentially interacting proteins in 4 enriched (FDR *q*-value < 0.10) Gene ontology biological process (GOBP) pathways that play a role in cell adhesion (Additional file 6). These pathways involved desmosomal proteins including DSG2, catenin alpha-1 (CTNA1), CDH1 and CDH3, and HER2 that is linked to proliferation regulation. The ability of parthenolide to alter protein–protein interactions of DSC1 with desmosomal proteins was demonstrated by negative enrichment of three GOBP pathways (Fig. 4).

Analysis of known interacting partners of DSC1 was performed in the STRING database. The interaction site of DSC1 (Fig. 3B) showed mainly interactions of DSC1 with proteins forming the desmosome structures (DSG2) and with proteins envoplakin (EVPL) and periplakin (PPL) that represent components of keratinocytes that contain copious desmosome structures. All proteins illustrated on the interaction map showed score > 0.950 that corresponds with high probability of correctness of these interactions.

In conclusion, pull-down analysis of DSC1 interacting proteins revealed DSC1 to interact not only with cytoskeletal proteins and proteins mediating cell adhesion, but also with proteins regulating cell proliferation, especially tyrosine kinase receptors HER2 and HER3, highlighting possible significant role of DSC1 in breast cancer progression. Moreover, parthenolide was found to modulate DSC1 interacting partners including protein HER3.

## Discussion

DSC1 is a transmembrane protein that maintains cell–cell adhesion as a part of desmosomes [23]. Previous studies suggested DSC1 to play a role in progression of various cancer diseases. For instance, DSC1 was found overexpressed in liver metastasis of colorectal tumors [24] and a lack of DSC1 protein in squamous cell carcinoma was associated with increased patient survival [25]. Moreover, DSC1 was found to positively influence β-catenin, c-myc and cyclin D1 signaling pathways leading to cancer progression [26]. Contradictory to these studies, others showed DSC1 protein to be negatively associated with disease progression. Low expression of desmocollins including DSC1 in colorectal carcinoma was related to higher tumor grade [27]. DSC1 was also overexpressed in primary melanoma and downregulated in melanoma metastases [28].

In the context of breast cancer, our previous study [13] revealed increased protein levels of DSC1 in luminal A breast tumors that invaded regional lymph nodes compared to lymph node negative luminal A breast tumors. Moreover, we confirmed DSC1 to increase migration and invasion of MCF7 breast cancer cell line in vitro [13]. Based on this evidence we suggested that DSC1 could be involved in metastatic mechanisms of luminal A breast tumors.

Herein, we firstly investigated DSC1 modulation in breast cancer. In our previous GSEA analysis of 836 primary breast tumor transcription profiles [11], we identified NF-κB pathway to be associated with positive lymph node status of luminal A patients in general and linked Cdk2 to the high risk of distant metastasis in lymph node negative luminal A patients. Based on these results we proposed a panel of inhibitors of these potential therapeutic targets that previously exhibited ability to suppress luminal A breast cancer in vitro and eventually in vivo. These inhibitors include NF-κB inhibitor parthenolide and Cdk2 inhibitor norcantharidin with which we tested additional NF-κB inhibitor niclosamide. Parthenolide inhibits MCF7 mammosphere formation and proliferation of MCF7 cells in vitro [29] and suppresses MCF7 mice xenografts [30]. Norcantharidin, clinically used drug for liver cancer treatment [31] was shown to repress breast cancer cell growth, adhesion, and migration and to induce apoptosis [32, 33]. Moreover, niclosamide, Food and Drug Administration-approved drug was found to induce inhibition of breast cancer cell growth [34, 35]. In the present study we tested these inhibitors to identify a modulator of DSC1 expression in luminal A breast cancer cells. We revealed that parthenolide significantly decreased DSC1 protein levels in MCF7 cells producing DSC1. Mechanism of parthenolide inhibition of desmosomal proteins is however not well understood to date. Nevertheless, previous studies [36–39] identified parthenolide to covalently bind several protein targets, including NF-κB factors and kinases associated with cancer development. Based on this evidence we conclude possible indirect inhibition of DSC1 by parthenolide.

AFM uncovered DSC1 over-expression in MCF7 cells to cause statistically significant decrease of cell height. This can be assumed as a pro-metastatic effect since reduced cell height was linked to metastatic phenotype [40]. Moreover, AFM revealed significant decrease of cell stiffness after parthenolide treatment in DSC1 overexpressing cells. AFM studies offered contradictory evidence on association between cell stiffness and metastatic potential. Some studies [41–43] showed cancer cells to be more elastic than normal cell lines and connected decreased cell stiffness to activation of EMT and invasion of cancer cells to distant tissue. Other studies [44–46] evidenced increased rigidity of cytoplasmatic membrane to be associated with higher pro-metastatic properties of cancer cells. Our results suggest that both DSC1 and parthenolide treatment influence morphology of MCF7 cell line.

To study molecular mechanisms associated with DSC1 overexpression and its inhibition by parthenolide, we performed analysis of the total proteome and transcriptome using DIA-LC–MS/MS and RNA-Seq, respectively. The most co-expressed genes with DSC1 modulated by parthenolide treatment were IGFBP5 and LACRT. IGFBP5 participates in cell growth, differentiation of human embryonic cells and in homeostasis in adult cells. Moreover, IGFBP5 can regulate IGF-II [47], apoptotic molecules (bax, bcl-2) [48] and p38 MAP kinase and Erk 1/2 signal transduction pathways [49], and thus influences cell proliferation, migration, survival, adhesion, and apoptosis [50–53]. Nevertheless, the reports of the role of IGFBP5 in cellular growth are contradictory, suggesting a complex role of IGFBP5 in cancer cells, as it can either stimulate or inhibit cell proliferation in various cell types [54]. IGFBP5 was revealed to stimulate cell migration in breast cancer [54]. Clinical observations provided supporting evidence that IGFBP5 is associated with metastasis and the aggressive tumor phenotype in breast cancer [55–58]. IGFBP5 was found overexpressed in breast tumors compared to normal breast tissues [59–61]. Hao et al. showed increased expression of IGFBP5 in lymph node metastases of breast carcinoma [57] and Li et al. demonstrated association between increased transcript levels of IGFBP5 and axillary lymph node metastasis and estrogen receptor expression in breast tumors [62]. Increased transcript levels of IGFBP5 also correlated with decreased survival of lymph node negative and estrogen receptor negative patients [62]. Contrary to these findings, IGFBP5 was found to inhibit growth of breast cancer cells in vitro and in vivo [48] and IGFBP5 overexpression was associated with improved breast cancer patient outcome in another study [63]. This inconsistency in suggestions of IGFBP5 role in breast cancer could be due to IGFBP5 protein cellular localization that seems to affect its ability to promote or inhibit breast cancer progression [64]. Tumor tissues mainly contain cytoplasmic IGFBP5, whereas cell lines with exogenously introduced IGFBP5 include mainly nuclear IGFBP5 acting as a growth inhibitor. Change of IGFBP5 localization from nucleus to cytoplasm switched IGFBP5 to a promoter of growth and migration [64]. This is in agreement with clinical observations indicating cytoplasmic IGFBP5 as a marker of poor prognosis [64]. LACRT is a glycoprotein that functions as a mitogen and promotes homeostasis and proliferation in human corneal epithelial cells [65, 66]. Mitogenic function of LACRT via NFAT and mTOR activation was observed in human embryonic kidney cells and human corneal epithelial cells [67]. LACRT was found expressed in normal breast tissue, breast tumors and breast cancer cell lines [68]. Amplification of LACRT gene was reported to be associated with invasion of breast tumors and was suggested as a marker for circulating breast cancer cells [69] which indicates connection of LACRT to metastatic behavior of breast tumors. These results suggest that DSC1-related molecular mechanisms supporting the lymph node metastasis development of luminal breast tumors could be associated with IGFBP5, known regulator of cell growth, migration, and proliferation, and with proliferation-promoting protein LACRT.

We further showed DSC1 overexpression to be associated with enrichment of MCM pathway that consists of CDK2 and MCM subunits 2–7. CDK2 binds to cyclin partners and promotes cell cycle progression [70, 71], whereas MCM2-7 complex acts as helicase and enables DNA replication during the S phase of the cell cycle [72]. Enrichment of this pathway supports the findings of association between DSC1 and proteins involved in proliferation in MCF7 breast cancer cells with potential to promote breast tumor aggressiveness and metastatic behavior.

Mechanisms of parthenolide-induced DSC1 inhibition include downregulation of IGFBP5 and LACRT together with proliferative proteins of MCM pathway. From these results we suggest that parthenolide treatment could target cellular mechanisms connected to DSC1 in luminal A breast cancer cells.

We have next focused on cellular mechanisms associated with DSC1 via protein–protein interactions. Previously known interaction partners of DSC1 include desmosomal proteins involved in cell adhesion and proteins related to epithelial cell phenotype. In concordance with main role of DSC1 in mediating cell adhesion through desmosome formation and interactions with desmosomal proteins [73, 74], potential DSC1 interacting proteins identified in our pull-down experiment involve mainly cadherins, namely DSG2, CDH1, CDH3 and several protocadherins. Cadherins typically participate in cell–cell adhesion, however cadherins are also known to interact with junctional proteins (such as β-catenin) and with growth factor receptors and thus can affect cell proliferation, motility, and survival [75]. CDH1 is a tumor suppressor that inhibits multiple steps of metastatic cascade [76]. CDH3 is a vital protein in maintenance of correct tissue architecture [77]. In breast cancer, overexpression of CDH3 is connected to more aggressive tumor behavior and poor patient survival [78, 79]. We found DSC1 to interact also with tyrosine kinase receptors HER2 and HER3 and parthenolide to modulate this DSC1-HER3 interaction. HER2 is an oncogene that serves as a very important clinical marker and therapeutic target in breast cancer [80]. HER3 is known to participate in oncogenic signaling through activation of the PI-3 K/Akt pathway and Src kinase, which induces cell proliferation and survival [81–83]. Further, HER3 is important for cell motility and enhances metastatic potential of breast tumor cells [84]. Increased expression of HER3 in multiple cancer diseases including breast cancer results in decreased patient survival [85] and treatment failures in cancer therapy [86–88]. Based on the results of this study supporting previous evidence of DSC1 role in breast cancer metastasis we conclude that DSC1 could participate in breast tumor progression by co-expression with genes involved in cell proliferation in the early stage of the disease, and support the formation of secondary tumors via physical interactions of DSC1 protein with proteins maintaining cellular adhesion in the later stage of the disease.

## Conclusions

We show association of DSC1, protein previously linked with lymph node metastasis of luminal A breast tumors, with increased metastatic potential of luminal A breast cancer cells in vitro. Although DSC1 is primarily involved in cell adhesion, proteomics and transcriptomics analysis reveal DSC1 to increase expression of IGFBP5 and LACRT and to positively regulate pathway of cell proliferation. Moreover, our results indicate potential regulation of DSC1 by NF-κB inhibitor parthenolide as we identified parthenolide to reduce DSC1 protein levels in MCF7 breast cancer cells as well as expression of IGFBP5, LACRT genes and proliferative pathway. Besides cell adhesion proteins as CDH1, CDH3 and DSG2, DSC1 interacts with tyrosine kinase receptors HER2 and HER3. Our data indicate that DSC1 is connected to cell migration, invasion, and cell cycle regulation in luminal A breast cancer cells, and can be effectively modulated by parthenolide. Based on these results we conclude that DSC1 could be involved in breast tumor proliferation and development in the early stage, and in tumor cell adhesion to metastatic sites in the later stage of the disease supporting generation of secondary tumors.

### Supplementary Information

**Additional file 1: Fig. S1.** Western blot analysis of DSC1 modulation using inhibitors niclosamide, norcantharidin and parthenolide. (A) processed images, (B) raw image of immunoblotting with anti-DSC1 antibody, (C) raw image of immunoblotting with anti-ACTB antibody. Tab. S1 Statistical evaluation of semiquantitative analysis of effect of inhibitors on protein levels of DSC1 longer and shorter isoforms. Fig. S2 (A) Representative chromatograms of iRT peptides measured in the total proteome experiment displayed in Skyline software, (B) iRT calibration chart used in the directDIA analysis in Spectronaut software. Tab. S2 Proteins significantly up-regulated after DSC1 overexpression. Fig. S3 Western blot verification of SBP tag presence in control MCF7-GFP cells and MCF7-DSC1-GFP cells used for pull-down identification of DSC1 protein interaction partners. Antibodies: Anti-SBP-tag (left), Anti-DSC1 (right) and Anti-ACTB (down).

**Additional file 2.** Total proteome experiment results. A) Mass spectrometry protein group level data from total proteome experiment comparing protein levels in MCF7-DSC1-GFP and control MCF7-GFP cell line. B) Mass spectrometry protein group level data from total proteome experiment comparing protein levels in MCF7-DSC1-GFP cell line treated with parthenolide and MCF7-DSC1-GFP cell line treated with DMSO. C) Mass spectrometry protein group level data from total proteome experiment comparing protein levels in MCF7-GFP cell line treated with parthenolide and MCF7-GFP cell line treated with DMSO. D) Mass spectrometry protein group level data from total proteome experiment comparing protein levels in MCF7-DSC1-GFP cell line treated with parthenolide and MCF7-GFP cell line treated with parthenolide.

**Additional file 3.** Results of RNA-Seq analysis of total transcriptome. A) RNA-Seq protein-coding transcript level data from transcriptomics experiment comparing transcript profiles in MCF7-DSC1-GFP and control MCF7-GFP cell line. B) RNA-Seq protein-coding transcript level data from transcriptomics experiment comparing transcript profiles in MCF7-DSC1-GFP cell line treated with parthenolide and MCF7-DSC1-GFP cell line treated with DMSO. C) RNA-Seq protein-coding transcript level data from transcriptomics experiment comparing transcript profiles in MCF7-GFP cell line treated with parthenolide and MCF7-GFP cell line treated with DMSO. D) RNA-Seq protein-coding transcript level data from transcriptomics experiment comparing transcript profiles in MCF7-DSC1-GFP cell line treated with parthenolide and MCF7-GFP cell line treated with parthenolide.

**Additional file 4.** Results of Gene Set Enrichment Analysis (GSEA) of A) total proteome data and B) transcriptomics data including GSEA enriched pathways and core enriched genes.

**Additional file 5.** Results of the pull-down identification of DSC1 protein interaction partners. A) Mass spectrometry protein group level data from pull-down experiment comparing protein levels in MCF7-DSC1-GFP and control MCF7-GFP cell line pull-downs. B) Mass spectrometry protein group level data from pull-down experiment comparing protein levels in MCF7-DSC1-GFP cell line treated with parthenolide and MCF7-DSC1-GFP treated with DMSO cell line pull-downs. C) Proteins significantly upregulated (*q*-value < 0.05 and Log2 FC > 1) in pull-down without parthenolide (PTL) treatment (comparison MCF7-DSC1-GFP vs. MCF7-GFP) and not statistically significantly upregulated in total proteome analysis (either "*q*-value > 0.05 when Log2 FC > 0" or "Log2 FC < 0" or not identified) in total proteome comparison MCF7-DSC1-GFP vs. MCF7-GFP.

**Additional file 6.** Results of Gene Set Enrichment Analysis (GSEA) of pull-down data including GSEA enriched pathways and core enriched genes.

## Data Availability

The raw mass spectrometry proteomics data and output files for the total proteome and pull-down analyses have been deposited in the ProteomeXchange Consortium via the Proteomics Identifications (PRIDE) partner repository (http://www.ebi.ac.uk/pride/archive/) with the dataset identifier PXD041029. The raw and processed RNA-Seq data have been deposited in NCBI's Gene Expression Omnibus [89] and are accessible through GEO Series accession number GSE228017 (https://www.ncbi.nlm.nih.gov/geo/query/acc.cgi?acc=GSE228017).

## Abbreviations

ACN: Acetonitrile
AFM: Atomic force microscopy
CDH1: Cadherin 1
CDH3: Cadherin 3
CDK2: Cyclin-dependent kinase 2
CTNA1: Catenin alpha-1
DIA: Data independent acquisition
DMEM: Dulbecco's Modified Eagle´s Medium
DMSO: Dimethyl sulfoxide
DSC1: Desmocollin-1
DSG2: Desmoglein 2
ECL: Enhanced chemiluminescence
EVPL: Envoplakin
FA: Formic acid
FASP: Filter-aided sample preparation
FBS: Fetal bovine serum
GFP: Green fluorescent protein
GOBP: Gene ontology biological process
GSEA: Gene set enrichment analysis
HCD: Higher-energy collisional dissociation
HER2: Receptor tyrosine-protein kinase erbB-2
HER3: Receptor tyrosine-protein kinase erbB-3
IGFBP5: Insulin-like growth factor-binding protein 5
LACRT: Extracellular glycoprotein lacritin
LC–MS/MS: Liquid chromatography-tandem mass spectrometry
MCM: Minichromosome maintenance
NF-κB: Nuclear factor kappa-light-chain-enhancer of activated B cells
PBS: Phosphate buffered saline
PPL: Periplakin
RNA-Seq: RNA sequencing
SBP: Streptavidin-binding peptide
