# Supplementary material for: Desmocollin-1 is associated with pro-metastatic phenotype of luminal A breast cancer cells and is modulated by parthenolide
Source: Cell Mol Biol Lett. 2023 Aug 24;28:68. doi: 10.1186/s11658-023-00481-6 (PMC10464112; doi:10.1186/s11658-023-00481-6)
Supplement: Supplementary file 1 — Additional file 1: Fig. S1. Western blot analysis of DSC1 modulation using inhibitors niclosamide, norcantharidin and parthenolide. (A) processed images, (B) raw image of immunoblotting with anti-DSC1 antibody, (C) raw image of immunoblotting with anti-ACTB antibody. Tab. S1 Statistical evaluation of semiquantitative analysis of effect of inhibitors on protein levels of DSC1 longer and shorter isoforms. Fig. S2 (A) Representative chromatograms of iRT peptides measured in the total proteome experiment displayed in Skyline software, (B) iRT calibration chart used in the directDIA analysis in Spectronaut software. Tab. S2 Proteins significantly up-regulated after DSC1 overexpression. Fig. S3 Western blot verification of SBP tag presence in control MCF7-GFP cells and MCF7-DSC1-GFP cells used for pull-down identification of DSC1 protein interaction partners. Antibodies: Anti-SBP-tag (left), Anti-DSC1 (right) and Anti-ACTB (down). [file 11658_2023_481_MOESM1_ESM.docx]

**Additional file 1: Tables and Figures**


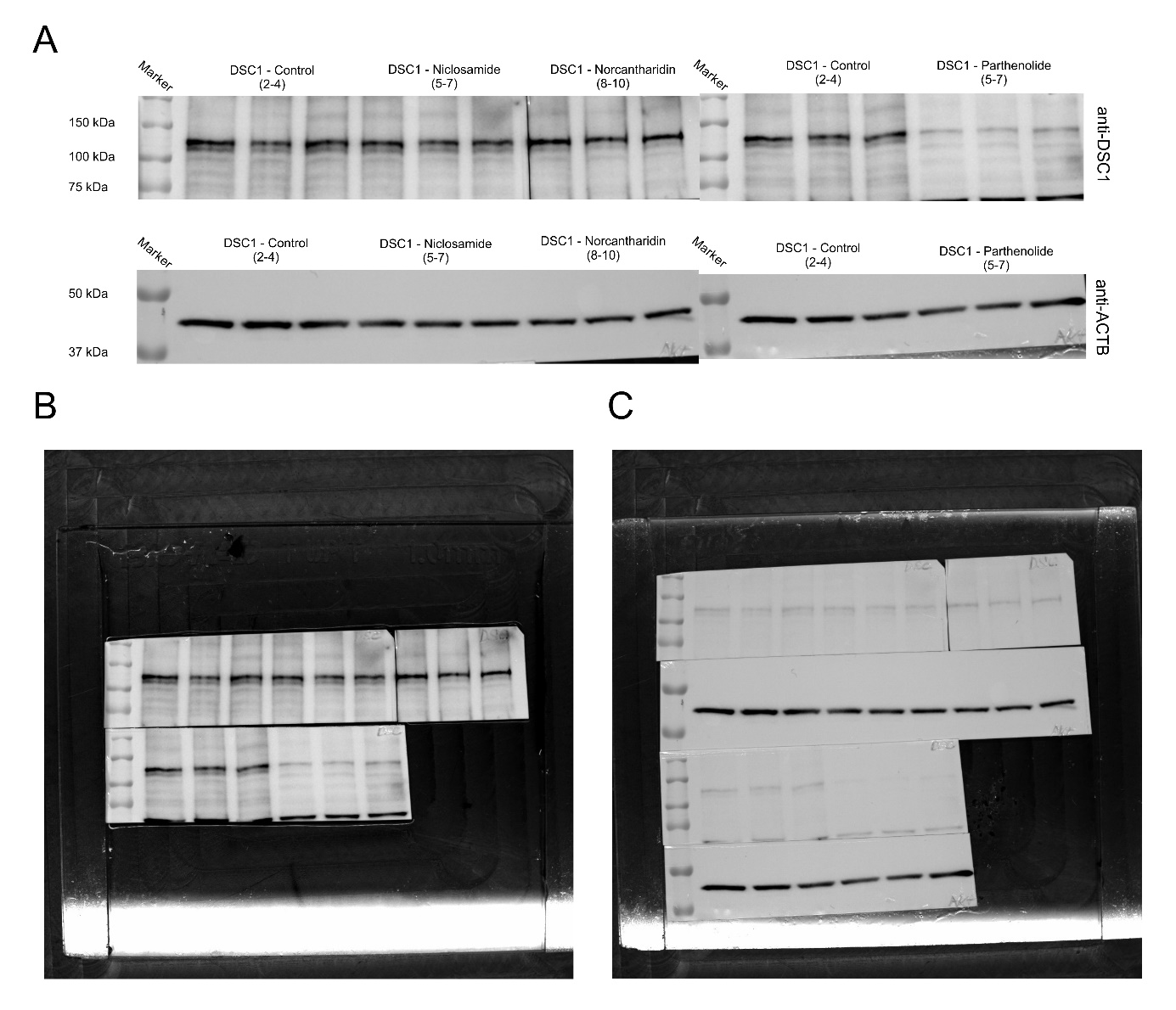


**Fig. S1** Western blot analysis of DSC1 modulation using inhibitors niclosamide, norcantharidin and parthenolide. (A) processed images, (B) raw image of immunoblotting with anti-DSC1 antibody, (C) raw image of immunoblotting with anti-ACTB antibody

**Table S1** Statistical evaluation of semiquantitative analysis of effect of inhibitors on protein levels of DSC1 longer and shorter isoforms.

| **Preprotein of DSC1 - longer isoform** | | | | | |
| --- | --- | --- | --- | --- | --- |
|  | Control | Niclosamide | Norcantharidin | Control | Parthenolide |
| DSC1 : ACTB ratio (1.-3. replicate) | 0.61 | 0.88 | 1.37 | 0.69 | 0.45 |
|  | 0.89 | 0.73 | 1.03 | 0.76 | 0.29 |
|  | 0.91 | 0.83 | 0.89 | 0.89 | 0.27 |
| Mean | 0.80 | 0.81 | 1.10 | 0.78 | 0.34 |
| Standard deviation | 0.1382 | 0.0624 | 0.1986 | 0.0823 | 0.0834 |
| **T-test** |  | **0.94** | **0.16** |  | **0.01** |
| Fold Change | 1 | 1.01 | 1.35 | 1 | 0.429 |
| **Active form of DSC1 - shorter isoform** | | | | | |
|  | Control | Niclosamide | Norcantharidin | Control | Parthenolide |
| DSC1 : ACTB ratio (1.-3. replicate) | 0.49 | 0.68 | 0.45 | 0.38 | 0.00 |
|  | 0.47 | 0.49 | 0.35 | 0.43 | 0.00 |
|  | 0.67 | 0.52 | 0.41 | 0.46 | 0.15 |
| Mean | 0.54 | 0.56 | 0.40 | 0.42 | 0.05 |
| Standard deviation | 0.1136 | 0.1006 | 0.0492 | 0.0409 | 0.0884 |
| **T-test** |  | **0.85** | **0.12** |  | **0.00** |
| Fold Change | 1 | 1.03 | 0.721 | 1 | 0.121 |


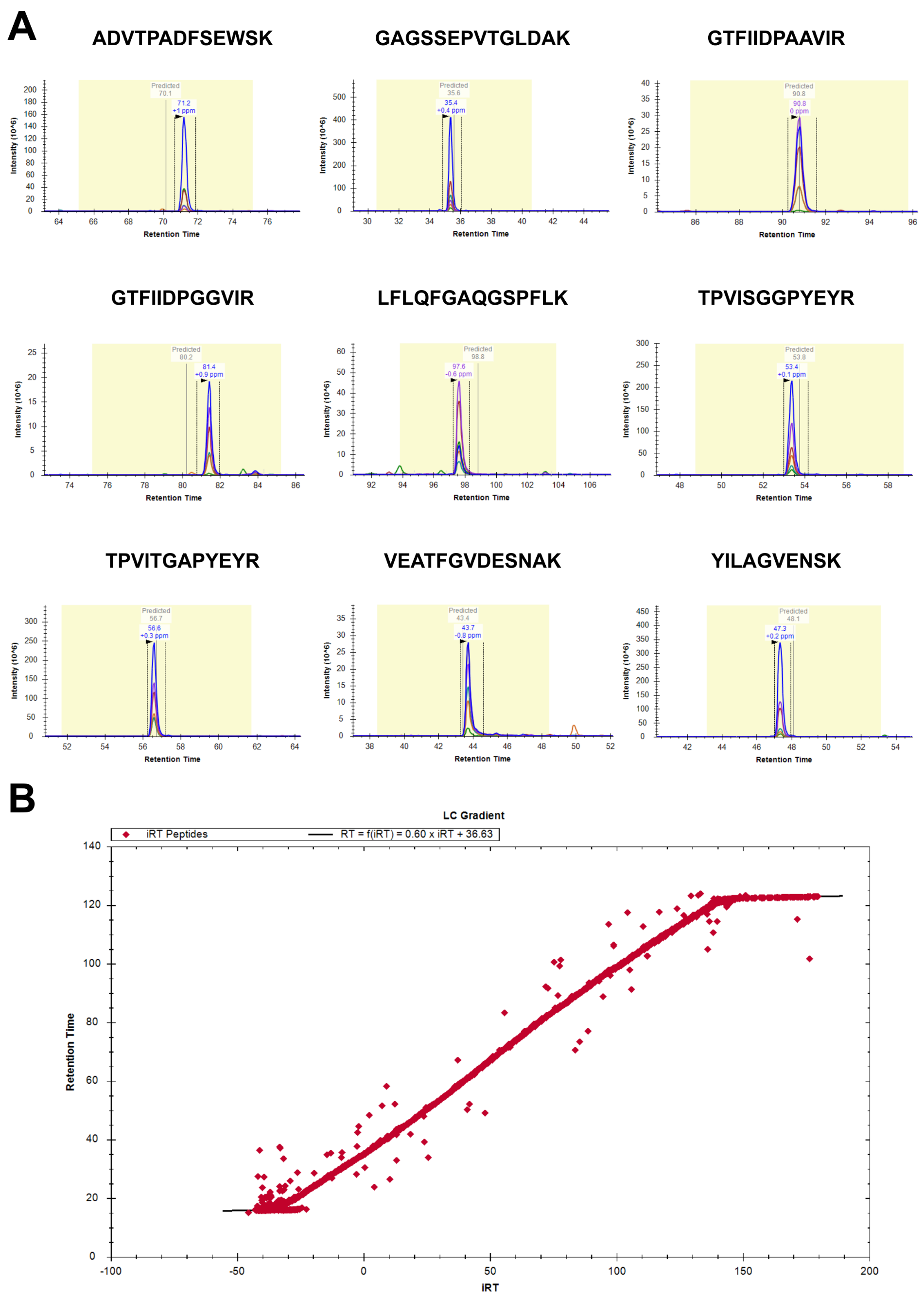


**Fig. S2** (A) Representative chromatograms of iRT peptides measured in the total proteome experiment displayed in Skyline software, (B) iRT calibration chart used in the directDIA analysis in Spectronaut software.

**Table S2** Proteins significantly up-regulated after DSC1 overexpression.

| **Gene name** | **UniProt ID** | **Protein description** | **MCF7-DSC1-GFP vs. MCF7-GFP** | | **MCF7-DSC1-GFP + PTL vs. MCF7-DSC1-GFP** | | **MCF7-GFP + PTL vs. MCF7-GFP** | | **MCF7-DSC1-GFP + PTL vs. MCF7-GFP + PTL** | |
| --- | --- | --- | --- | --- | --- | --- | --- | --- | --- | --- |
|  |  |  | **log2 FC** | **q-value** | **log2 FC** | **q-value** | **log2 FC** | **q-value** | **log2 FC** | **q-value** |
| DSC1 | Q08554 | Desmocollin-1 | 4.07 | 5.53E-49 | -0.52 | 3.31E-28 | -1.19 | 0.025 | 4.74 | 3.17E-40 |
| LACRT | Q9GZZ8 | Extracellular glycoprotein lacritin | 3.79 | 1.96E-03 | -2.62 | 0.025 | 0.33 | 0.238 | 0.84 | 0.174 |
| IGFBP5 | P24593 | Insulin-like growth factor-binding protein 5 | 2.87 | 2.92E-04 | -2.50 | 0.014 | -0.99 | 0.043 | 1.37 | 0.090 |
| IGLL5 | B9A064 | Immunoglobulin lambda-like polypeptide 5 | 2.77 | 4.10E-03 | -1.42 | 0.057 | 1.75 | 0.069 | -0.40 | 0.223 |
| IGHA1 | P01876 | Immunoglobulin heavy constant alpha 1 | 2.66 | 0.014 | -3.17 | 0.025 | -1.12 | 0.267 | 0.61 | 0.195 |
| MYO5C | Q9NQX4 | Unconventional myosin-Vc | 2.59 | 1.10E-03 | -0.36 | 0.010 | -0.56 | 0.138 | 2.79 | 3.51E-03 |
| SPPL2A | Q8TCT8 | Signal peptide peptidase-like 2A | 2.55 | 3.83E-04 | -0.21 | 0.141 | -0.46 | 0.073 | 2.81 | 1.83E-03 |
| HIST2H2AC | Q16777 | Histone H2A type 2-C | 2.42 | 6.18E-04 | -0.83 | 0.197 | -1.37 | 0.051 | 2.96 | 1.61E-06 |
| SLC27A2 | O14975 | Very long-chain acyl-CoA synthetase | 2.37 | 1.17E-08 | -0.55 | 7.60E-05 | -0.88 | 1.43E-03 | 2.70 | 2.08E-07 |
| IGKC | P01834 | Immunoglobulin kappa constant | 2.25 | 0.025 | -1.14 | 0.150 | 0.24 | 0.192 | 0.86 | 0.041 |
| PDCD4 | Q53EL6 | Programmed cell death protein 4 | 2.22 | 6.88E-12 | -1.71 | 1.05E-13 | 0.04 | 0.063 | 0.47 | 1.45E-04 |
| USP8 | P40818 | Ubiquitin carboxyl-terminal hydrolase 8 | 2.21 | 7.99E-07 | 0.24 | 5.70E-05 | 0.45 | 0.016 | 2.00 | 3.92E-11 |
| SFXN2 | Q96NB2 | Sideroflexin-2 | 1.65 | 7.95E-14 | -0.34 | 5.54E-05 | -0.72 | 2.89E-06 | 2.03 | 6.33E-11 |
| TSPAN13 | O95857 | Tetraspanin-13 | 1.47 | 2.76E-04 | 0.00 | 0.285 | 0.03 | 0.210 | 1.43 | 1.44E-05 |
| FREM2 | Q5SZK8 | FRAS1-related extracellular matrix protein 2 | 1.44 | 2.20E-14 | 2.28 | 1.15E-06 | 3.59 | 4.59E-03 | 0.14 | 0.133 |
| GFRA1 | P56159 | GDNF family receptor alpha-1 | 1.34 | 4.51E-15 | -2.17 | 2.65E-16 | -1.56 | 1.67E-12 | 0.73 | 2.95E-05 |
| PPP1R3D | O95685 | Protein phosphatase 1 regulatory subunit 3D | 1.31 | 5.21E-08 | -0.72 | 2.11E-05 | -0.51 | 0.011 | 1.10 | 2.70E-03 |
| ANXA6 | P08133 | Annexin A6 | 1.29 | 1.34E-18 | -0.26 | 5.17E-03 | 0.16 | 6.71E-03 | 0.87 | 8.25E-14 |
| SELENOS | Q9BQE4 | Selenoprotein S | 1.27 | 0.023 | 0.13 | 0.212 | -0.73 | 0.057 | 2.13 | 0.011 |
| NR3C1 | P04150 | Glucocorticoid receptor | 1.26 | 3.02E-03 | 0.01 | 0.267 | 0.41 | 0.024 | 0.86 | 5.61E-03 |
| RHOB | P62745 | Rho-related GTP-binding protein RhoB | 1.24 | 1.18E-07 | -0.55 | 1.26E-06 | 0.12 | 0.162 | 0.57 | 2.52E-04 |
| RAB31 | Q13636 | Ras-related protein Rab-31 | 1.22 | 0.016 | -0.23 | 0.011 | -0.17 | 0.256 | 1.15 | 1.26E-03 |
| H1FX | Q92522 | Histone H1x | 1.21 | 5.29E-07 | -1.21 | 1.82E-03 | -0.89 | 0.023 | 0.89 | 0.032 |
| NEDD4L | Q96PU5 | E3 ubiquitin-protein ligase NEDD4-like | 1.19 | 0.022 | 0.03 | 0.272 | 0.22 | 0.029 | 1.00 | 0.036 |
| RCN1 | Q15293 | Reticulocalbin-1 | 1.18 | 1.65E-15 | -0.05 | 0.075 | -0.23 | 7.62E-05 | 1.36 | 1.19E-18 |
| COX11 | Q9Y6N1 | Cytochrome c oxidase assembly protein COX11, mitochondrial | 1.15 | 1.33E-03 | -1.01 | 4.00E-04 | -1.58 | 2.98E-04 | 1.71 | 1.57E-03 |
| HIST1H1E | P10412 | Histone H1.4 | 1.10 | 6.26E-15 | -0.63 | 9.07E-06 | -0.69 | 2.76E-13 | 1.16 | 6.11E-19 |
| ERGIC1 | Q969X5 | Endoplasmic reticulum-Golgi intermediate compartment protein 1 | 1.10 | 7.95E-16 | -0.34 | 7.17E-07 | -0.68 | 2.26E-07 | 1.43 | 8.34E-15 |
| TMOD3 | Q9NYL9 | Tropomodulin-3 | 1.06 | 8.46E-16 | 0.16 | 1.42E-03 | 0.29 | 2.09E-04 | 0.93 | 2.21E-14 |
| UAP1L1 | Q3KQV9 | UDP-N-acetylhexosamine pyrophosphorylase-like protein 1 | 1.03 | 3.00E-04 | -0.02 | 0.257 | 0.50 | 5.38E-03 | 0.51 | 0.010 |
| TOM1L1 | O75674 | TOM1-like protein 1 | 1.03 | 1.01E-06 | 0.11 | 7.91E-03 | 0.18 | 0.039 | 0.95 | 1.38E-04 |
| F5 | P12259 | Coagulation factor V | 1.00 | 9.72E-12 | -0.25 | 1.45E-03 | -0.07 | 0.288 | 0.82 | 5.14E-05 |
| RBM12B | Q8IXT5 | RNA-binding protein 12B | 0.98 | 0.023 | -0.06 | 0.040 | -0.07 | 0.070 | 0.99 | 1.07E-04 |
| BCS1L | Q9Y276 | Mitochondrial chaperone BCS1 | 0.98 | 2.88E-06 | -0.28 | 0.040 | -0.48 | 5.56E-04 | 1.17 | 1.37E-07 |
| MREG | Q8N565 | Melanoregulin | 0.97 | 0.014 | -0.62 | 0.059 | -1.12 | 0.025 | 1.47 | 8.44E-03 |
| TMEM164 | Q5U3C3 | Transmembrane protein 164 | 0.96 | 5.79E-04 | -0.60 | 8.54E-03 | -1.04 | 2.57E-03 | 1.40 | 4.43E-04 |
| KIF23 | Q02241 | Kinesin-like protein KIF23 | 0.96 | 3.68E-05 | -2.25 | 7.23E-07 | -1.56 | 1.50E-03 | 0.27 | 0.222 |
| FKBP10 | Q96AY3 | Peptidyl-prolyl cis-trans isomerase FKBP10 | 0.95 | 0.013 | -0.46 | 0.021 | -0.67 | 5.26E-04 | 1.17 | 0.017 |
| HIST1H1B | P16401 | Histone H1.5 | 0.95 | 6.93E-11 | -0.54 | 1.35E-06 | -0.71 | 3.34E-07 | 1.13 | 1.17E-14 |
| H1F0 | P07305 | Histone H1.0 | 0.95 | 3.36E-07 | -0.60 | 7.67E-04 | -0.73 | 3.70E-06 | 1.08 | 1.52E-08 |
| CKMT1A | P12532 | Creatine kinase U-type, mitochondrial | 0.94 | 7.41E-07 | -0.41 | 2.53E-03 | -0.67 | 1.37E-06 | 1.20 | 8.29E-09 |
| RACGAP1 | Q9H0H5 | Rac GTPase-activating protein 1 | 0.93 | 0.022 | -2.47 | 0.071 | -1.40 | 0.062 | -0.15 | 0.183 |
| FAM160B1 | Q5W0V3 | Protein FAM160B1 | 0.92 | 4.94E-05 | -0.02 | 0.210 | 0.36 | 5.09E-03 | 0.53 | 5.04E-04 |
| NUMA1 | Q14980 | Nuclear mitotic apparatus protein 1 | 0.90 | 2.95E-61 | -0.96 | 3.32E-69 | -1.20 | 1.29E-59 | 1.13 | 1.01E-37 |
| CDV3 | Q9UKY7 | Protein CDV3 homolog | 0.89 | 7.83E-14 | -0.14 | 0.017 | 0.53 | 6.15E-09 | 0.22 | 0.157 |
| LXN | Q9BS40 | Latexin | 0.86 | 2.51E-12 | -0.33 | 1.39E-03 | 0.20 | 0.020 | 0.34 | 8.17E-06 |
| PSIP1 | O75475 | PC4 and SFRS1-interacting protein | 0.86 | 1.07E-09 | -0.76 | 3.38E-09 | -0.82 | 9.52E-07 | 0.92 | 4.32E-10 |
| MACROD1 | Q9BQ69 | ADP-ribose glycohydrolase MACROD1 | 0.86 | 1.45E-10 | -0.65 | 6.11E-09 | -0.70 | 2.34E-06 | 0.91 | 3.55E-07 |
| OAT | P04181 | Ornithine aminotransferase, mitochondrial | 0.86 | 4.26E-09 | 0.38 | 1.26E-05 | 0.13 | 0.249 | 1.10 | 5.86E-13 |
| GHITM | Q9H3K2 | Growth hormone-inducible transmembrane protein | 0.86 | 1.58E-09 | 0.27 | 5.10E-03 | -0.07 | 0.165 | 1.20 | 5.41E-11 |
| MYO5A | Q9Y4I1 | Unconventional myosin-Va | 0.85 | 7.99E-05 | -0.06 | 0.125 | -0.38 | 0.023 | 1.18 | 2.42E-04 |
| MYOF | Q9NZM1 | Myoferlin | 0.84 | 3.78E-122 | -0.22 | 1.30E-15 | -0.59 | 1.25E-43 | 1.21 | 3.71E-93 |
| LMCD1 | Q9NZU5 | LIM and cysteine-rich domains protein 1 | 0.83 | 1.40E-03 | 0.26 | 8.84E-03 | 0.59 | 1.57E-03 | 0.51 | 0.011 |
| NUP54 | Q7Z3B4 | Nucleoporin p54 | 0.83 | 0.038 | -0.22 | 2.46E-03 | 0.06 | 0.160 | 0.54 | 0.047 |
| ZNF512B | Q96KM6 | Zinc finger protein 512B | 0.82 | 2.03E-03 | -2.68 | 1.15E-05 | -2.22 | 1.28E-03 | 0.36 | 0.018 |
| PPM1H | Q9ULR3 | Protein phosphatase 1H | 0.82 | 3.75E-04 | -0.22 | 0.158 | -0.07 | 0.202 | 0.67 | 7.87E-03 |
| HIST2H2AB | Q8IUE6 | Histone H2A type 2-B | 0.80 | 2.48E-04 | -0.43 | 0.029 | -0.78 | 2.70E-04 | 1.15 | 1.83E-04 |
| KIAA1324 | Q6UXG2 | UPF0577 protein KIAA1324 | 0.80 | 3.49E-09 | -1.51 | 9.52E-11 | -1.94 | 4.17E-05 | 1.23 | 4.78E-03 |
| GGCT | O75223 | Gamma-glutamylcyclotransferase | 0.80 | 2.76E-13 | -0.08 | 0.066 | 0.39 | 0.068 | 0.32 | 2.26E-04 |
| HIST1H3A | P68431 | Histone H3.1 | 0.79 | 3.71E-04 | -0.79 | 1.13E-03 | -0.90 | 0.023 | 0.90 | 6.70E-03 |
| APOH | P02749 | Beta-2-glycoprotein 1 | 0.79 | 1.36E-04 | -0.38 | 0.030 | -0.31 | 0.068 | 0.72 | 5.76E-03 |
| SQOR | Q9Y6N5 | Sulfide:quinone oxidoreductase, mitochondrial | 0.79 | 1.03E-10 | -0.10 | 0.050 | -0.45 | 1.02E-04 | 1.13 | 1.55E-14 |
| ECI1 | P42126 | Enoyl-CoA delta isomerase 1, mitochondrial | 0.78 | 2.03E-10 | -0.33 | 3.26E-03 | -0.85 | 1.09E-05 | 1.29 | 3.79E-06 |
| NRCAM | Q92823 | Neuronal cell adhesion molecule | 0.78 | 2.91E-03 | -1.32 | 6.13E-06 | -1.34 | 1.37E-04 | 0.79 | 0.010 |
| CWF19L1 | Q69YN2 | CWF19-like protein 1 | 0.77 | 0.010 | -1.01 | 0.032 | -1.01 | 0.019 | 0.77 | 0.073 |
| BCAM | P50895 | Basal cell adhesion molecule | 0.77 | 3.17E-13 | -0.48 | 3.57E-13 | -0.75 | 1.82E-10 | 1.03 | 1.86E-11 |
| HIST1H2AB | P04908 | Histone H2A type 1-B/E | 0.76 | 1.23E-03 | -0.48 | 0.162 | -0.72 | 0.258 | 1.00 | 5.20E-05 |
| PHGDH | O43175 | D-3-phosphoglycerate dehydrogenase | 0.76 | 2.97E-20 | -0.30 | 1.08E-10 | 0.01 | 0.158 | 0.45 | 1.52E-09 |
| ZCCHC8 | Q6NZY4 | Zinc finger CCHC domain-containing protein 8 | 0.75 | 7.14E-04 | -0.14 | 0.126 | -0.07 | 0.205 | 0.68 | 3.65E-03 |
| DNPEP | Q9ULA0 | Aspartyl aminopeptidase | 0.75 | 4.08E-07 | -0.21 | 0.014 | 0.04 | 0.211 | 0.49 | 4.23E-11 |
| H2AFZ | P0C0S5 | Histone H2A.Z | 0.74 | 1.22E-04 | -0.67 | 0.048 | -0.99 | 1.33E-03 | 1.07 | 6.44E-05 |
| TSTD1 | Q8NFU3 | Thiosulfate:glutathione sulfurtransferase | 0.74 | 6.93E-04 | -0.28 | 0.026 | 0.21 | 0.011 | 0.26 | 2.13E-03 |
| NR2F2 | P24468 | COUP transcription factor 2 | 0.74 | 3.50E-05 | -2.00 | 1.81E-05 | -1.84 | 2.92E-04 | 0.58 | 0.043 |
| HIST2H3A | Q71DI3 | Histone H3.2 | 0.73 | 1.34E-13 | -0.49 | 7.31E-06 | -0.63 | 6.85E-14 | 0.87 | 2.61E-14 |
| STMN1 | P16949 | Stathmin | 0.73 | 4.59E-04 | -0.58 | 1.43E-04 | 0.21 | 0.055 | -0.05 | 0.119 |
| HMGB1 | P09429 | High mobility group protein B1 | 0.73 | 5.70E-16 | -0.30 | 1.13E-03 | 0.07 | 0.195 | 0.36 | 3.51E-05 |
| SPRYD4 | Q8WW59 | SPRY domain-containing protein 4 | 0.72 | 6.86E-09 | 0.03 | 0.127 | -0.25 | 0.035 | 1.00 | 4.83E-11 |
| GLUD1 | P00367 | Glutamate dehydrogenase 1, mitochondrial | 0.72 | 1.37E-10 | -0.10 | 0.247 | -0.28 | 2.01E-06 | 0.90 | 1.62E-17 |
| IQGAP3 | Q86VI3 | Ras GTPase-activating-like protein IQGAP3 | 0.72 | 6.58E-04 | -2.11 | 1.24E-04 | -0.94 | 0.041 | -0.45 | 0.068 |
| NNT | Q13423 | NAD(P) transhydrogenase, mitochondrial | 0.71 | 5.42E-24 | -0.25 | 4.57E-07 | -0.72 | 4.50E-10 | 1.18 | 1.33E-27 |
| HIST1H2BK | O60814 | Histone H2B type 1-K | 0.71 | 5.99E-16 | -0.46 | 4.52E-09 | -0.67 | 5.17E-09 | 0.92 | 2.32E-13 |
| MTX2 | O75431 | Metaxin-2 | 0.71 | 0.025 | -0.32 | 0.046 | -0.53 | 1.51E-03 | 0.92 | 4.14E-03 |
| HIBADH | P31937 | 3-hydroxyisobutyrate dehydrogenase, mitochondrial | 0.71 | 4.72E-11 | -0.12 | 7.85E-03 | -0.46 | 9.02E-05 | 1.05 | 4.58E-08 |
| ANP32E | Q9BTT0 | Acidic leucine-rich nuclear phosphoprotein 32 family member E | 0.71 | 8.55E-06 | -0.51 | 2.07E-04 | 0.13 | 0.140 | 0.07 | 0.051 |
| TOP2B | Q02880 | DNA topoisomerase 2-beta | 0.71 | 2.98E-67 | -0.59 | 1.08E-28 | -0.78 | 2.99E-54 | 0.90 | 2.01E-44 |
| H3F3A | P84243 | Histone H3.3 | 0.71 | 2.07E-03 | -0.36 | 0.077 | -0.58 | 2.26E-04 | 0.93 | 8.61E-04 |
| PKIB | Q9C010 | cAMP-dependent protein kinase inhibitor beta | 0.71 | 0.027 | -0.41 | 0.130 | 0.03 | 0.286 | 0.27 | 0.182 |
| RMND1 | Q9NWS8 | Required for meiotic nuclear division protein 1 homolog | 0.71 | 3.69E-09 | -0.42 | 2.33E-06 | -0.75 | 3.25E-09 | 1.03 | 9.42E-12 |
| DUT | P33316 | Deoxyuridine 5'-triphosphate nucleotidohydrolase, mitochondrial | 0.70 | 0.023 | -0.31 | 0.283 | 0.17 | 6.86E-03 | 0.22 | 7.20E-04 |
| PDS5B | Q9NTI5 | Sister chromatid cohesion protein PDS5 homolog B | 0.69 | 1.25E-22 | -0.34 | 1.84E-12 | -0.55 | 6.84E-11 | 0.91 | 6.38E-17 |
| FBP2 | O00757 | Fructose-1,6-bisphosphatase isozyme 2 | 0.69 | 2.68E-03 | -0.24 | 0.029 | 0.25 | 0.029 | 0.20 | 0.027 |
| PCK2 | Q16822 | Phosphoenolpyruvate carboxykinase [GTP], mitochondrial | 0.69 | 4.70E-30 | -0.30 | 2.10E-12 | -0.51 | 6.81E-17 | 0.90 | 1.52E-28 |
| POLA2 | Q14181 | DNA polymerase alpha subunit B | 0.69 | 0.022 | -1.33 | 0.011 | -0.95 | 4.30E-03 | 0.30 | 0.044 |
| ABCB6 | Q9NP58 | ATP-binding cassette sub-family B member 6, mitochondrial | 0.68 | 1.85E-08 | -0.12 | 0.156 | -0.20 | 1.10E-03 | 0.76 | 9.78E-07 |
| HIBCH | Q6NVY1 | 3-hydroxyisobutyryl-CoA hydrolase, mitochondrial | 0.68 | 2.02E-09 | 0.27 | 5.98E-04 | -0.10 | 0.012 | 1.04 | 3.28E-11 |
| PREX1 | Q8TCU6 | Phosphatidylinositol 3,4,5-trisphosphate-dependent Rac exchanger 1 protein | 0.68 | 5.86E-25 | -1.23 | 7.63E-30 | -0.51 | 3.85E-13 | -0.04 | 0.042 |
| SFXN3 | Q9BWM7 | Sideroflexin-3 | 0.67 | 0.016 | -0.05 | 0.166 | -0.53 | 0.102 | 1.15 | 0.027 |
| BAZ1B | Q9UIG0 | Tyrosine-protein kinase BAZ1B | 0.67 | 1.34E-06 | -0.56 | 2.45E-05 | -0.96 | 6.21E-05 | 1.07 | 1.99E-05 |
| NHLRC2 | Q8NBF2 | NHL repeat-containing protein 2 | 0.67 | 0.011 | -0.48 | 0.024 | -0.54 | 0.139 | 0.73 | 0.120 |
| CRYBG1 | Q9Y4K1 | Beta/gamma crystallin domain-containing protein 1 | 0.67 | 7.99E-08 | -1.42 | 6.61E-09 | -1.34 | 1.30E-07 | 0.59 | 4.50E-03 |
| AQR | O60306 | RNA helicase aquarius | 0.66 | 7.15E-03 | -0.14 | 0.169 | -0.58 | 0.015 | 1.10 | 2.86E-05 |
| LNPK | Q9C0E8 | Endoplasmic reticulum junction formation protein lunapark | 0.66 | 1.79E-06 | -0.13 | 0.121 | -0.25 | 0.046 | 0.79 | 3.22E-09 |
| IRF2BPL | Q9H1B7 | Probable E3 ubiquitin-protein ligase IRF2BPL | 0.66 | 0.032 | -0.52 | 0.023 | -0.53 | 0.042 | 0.67 | 0.028 |
| PACSIN3 | Q9UKS6 | Protein kinase C and casein kinase substrate in neurons protein 3 | 0.66 | 3.29E-04 | -0.41 | 0.034 | -0.44 | 0.039 | 0.69 | 0.060 |
| CAV1 | Q03135 | Caveolin-1 | 0.66 | 3.16E-03 | -0.90 | 0.032 | -0.33 | 0.102 | 0.08 | 0.250 |
| GSTO1 | P78417 | Glutathione S-transferase omega-1 | 0.66 | 7.36E-09 | -0.02 | 0.217 | 0.32 | 4.76E-04 | 0.32 | 0.070 |
| GARS | P41250 | Glycine--tRNA ligase | 0.65 | 3.28E-16 | 0.57 | 3.78E-18 | 0.66 | 1.29E-20 | 0.56 | 2.24E-26 |
| NUDT1 | P36639 | 7,8-dihydro-8-oxoguanine triphosphatase | 0.65 | 4.82E-04 | 0.09 | 0.062 | 0.73 | 2.43E-04 | 0.01 | 0.259 |
| PBRM1 | Q86U86 | Protein polybromo-1 | 0.65 | 8.17E-03 | -0.47 | 0.060 | -0.99 | 2.37E-03 | 1.17 | 1.98E-03 |
| ERLIN1 | O75477 | Erlin-1 | 0.65 | 1.71E-04 | -0.32 | 0.014 | -0.57 | 1.40E-04 | 0.90 | 1.38E-04 |
| GTF3C4 | Q9UKN8 | General transcription factor 3C polypeptide 4 | 0.65 | 1.39E-04 | -0.64 | 8.72E-05 | -0.75 | 1.96E-05 | 0.76 | 5.58E-04 |
| ALDH4A1 | P30038 | Delta-1-pyrroline-5-carboxylate dehydrogenase, mitochondrial | 0.65 | 8.35E-12 | -0.28 | 2.16E-04 | -0.41 | 7.55E-11 | 0.78 | 1.53E-12 |
| KIDINS220 | Q9ULH0 | Kinase D-interacting substrate of 220 kDa | 0.64 | 0.023 | -0.40 | 0.073 | -0.65 | 1.23E-03 | 0.90 | 7.22E-04 |
| NIF3L1 | Q9GZT8 | NIF3-like protein 1 | 0.64 | 4.60E-05 | 0.03 | 0.192 | 0.41 | 5.21E-03 | 0.27 | 0.010 |
| ARFGEF3 | Q5TH69 | Brefeldin A-inhibited guanine nucleotide-exchange protein 3 | 0.64 | 7.97E-03 | -0.05 | 0.207 | 0.00 | 0.276 | 0.58 | 0.029 |
| BRD2 | P25440 | Bromodomain-containing protein 2 | 0.64 | 0.034 | 0.37 | 1.77E-03 | 0.27 | 0.113 | 0.74 | 6.24E-04 |
| SPIN1 | Q9Y657 | Spindlin-1 | 0.64 | 0.042 | -0.14 | 0.180 | -0.05 | 0.254 | 0.54 | 0.057 |
| IFI35 | P80217 | Interferon-induced 35 kDa protein | 0.63 | 0.034 | -0.75 | 0.100 | -0.23 | 0.175 | 0.11 | 0.286 |
| PI4K2A | Q9BTU6 | Phosphatidylinositol 4-kinase type 2-alpha | 0.63 | 1.11E-07 | 0.58 | 2.15E-07 | 0.38 | 1.85E-03 | 0.83 | 3.80E-07 |
| MYADM | Q96S97 | Myeloid-associated differentiation marker | 0.63 | 5.60E-07 | 0.38 | 5.94E-03 | 0.42 | 0.075 | 0.59 | 2.00E-06 |
| GTF3C2 | Q8WUA4 | General transcription factor 3C polypeptide 2 | 0.63 | 2.72E-03 | -0.60 | 3.86E-05 | -0.66 | 3.57E-04 | 0.68 | 1.53E-03 |
| SPRYD7 | Q5W111 | SPRY domain-containing protein 7 | 0.62 | 4.82E-04 | 0.31 | 3.42E-03 | 0.36 | 4.57E-03 | 0.58 | 2.02E-04 |
| TMPO | P42166 | Lamina-associated polypeptide 2, isoform alpha | 0.62 | 5.77E-12 | -0.55 | 2.59E-20 | -0.98 | 1.65E-05 | 1.01 | 8.54E-07 |
| CD63 | P08962 | CD63 antigen | 0.62 | 4.23E-05 | 0.17 | 0.224 | -0.14 | 0.149 | 0.93 | 2.08E-06 |
| LTF | P02788 | Lactotransferrin | 0.62 | 0.028 | -0.15 | 0.041 | -0.26 | 0.142 | 0.72 | 0.095 |
| SIN3A | Q96ST3 | Paired amphipathic helix protein Sin3a | 0.62 | 6.78E-08 | -0.54 | 3.36E-08 | -0.52 | 2.37E-08 | 0.60 | 6.39E-09 |
| TPBG | Q13641 | Trophoblast glycoprotein | 0.62 | 4.44E-05 | -0.03 | 0.222 | 0.13 | 0.167 | 0.46 | 5.39E-03 |
| SMPDL3B | Q92485 | Acid sphingomyelinase-like phosphodiesterase 3b | 0.61 | 2.38E-04 | -0.73 | 8.35E-04 | -1.00 | 2.33E-04 | 0.89 | 1.36E-03 |
| AFP | P02771 | Alpha-fetoprotein | 0.61 | 0.017 | -0.03 | 0.162 | -0.12 | 0.274 | 0.70 | 0.019 |
| SLC4A1AP | Q9BWU0 | Kanadaptin | 0.61 | 2.75E-07 | 0.50 | 2.42E-07 | 0.46 | 9.84E-08 | 0.65 | 9.32E-08 |
| DIABLO | Q9NR28 | Diablo homolog, mitochondrial | 0.61 | 3.02E-10 | -0.07 | 0.172 | -0.38 | 1.29E-09 | 0.92 | 2.27E-12 |
| C12orf10 | Q9HB07 | UPF0160 protein MYG1, mitochondrial | 0.61 | 6.43E-11 | -0.38 | 2.14E-06 | -0.01 | 0.261 | 0.24 | 3.72E-06 |
| HMGB2 | P26583 | High mobility group protein B2 | 0.61 | 9.03E-06 | -0.41 | 6.28E-03 | -0.14 | 0.024 | 0.34 | 5.48E-03 |
| PPP1R18 | Q6NYC8 | Phostensin | 0.61 | 1.69E-03 | 0.41 | 0.025 | 0.00 | 0.279 | 1.01 | 4.57E-03 |
| PRCP | P42785 | Lysosomal Pro-X carboxypeptidase | 0.60 | 2.13E-08 | -0.17 | 3.61E-04 | -0.54 | 9.94E-06 | 0.97 | 1.09E-08 |
| FHL1 | Q13642 | Four and a half LIM domains protein 1 | 0.60 | 5.67E-05 | -0.12 | 0.075 | 0.36 | 2.71E-03 | 0.12 | 0.096 |
| TXNRD2 | Q9NNW7 | Thioredoxin reductase 2, mitochondrial | 0.60 | 5.17E-04 | -0.36 | 3.28E-04 | -0.72 | 1.06E-04 | 0.96 | 2.81E-04 |
| ISOC1 | Q96CN7 | Isochorismatase domain-containing protein 1 | 0.60 | 5.92E-04 | -0.28 | 0.039 | 0.06 | 0.220 | 0.26 | 0.142 |
| CFAP100 | Q494V2 | Cilia- and flagella-associated protein 100 | 0.60 | 4.01E-03 | -0.07 | 0.243 | -0.11 | 0.149 | 0.64 | 1.70E-03 |
| IDH3A | P50213 | Isocitrate dehydrogenase [NAD] subunit alpha, mitochondrial | 0.59 | 1.45E-04 | 0.06 | 0.043 | -0.25 | 0.028 | 0.90 | 1.12E-18 |
| GDAP1 | Q8TB36 | Ganglioside-induced differentiation-associated protein 1 | 0.59 | 3.14E-05 | -0.15 | 0.033 | -0.61 | 2.78E-05 | 1.04 | 9.23E-07 |
| APOE | P02649 | Apolipoprotein E | 0.59 | 0.021 | 0.12 | 0.179 | 0.25 | 0.121 | 0.46 | 0.066 |
| BCCIP | Q9P287 | BRCA2 and CDKN1A-interacting protein | 0.59 | 2.59E-08 | -0.21 | 0.024 | 0.05 | 0.275 | 0.33 | 3.80E-04 |
| GLUD2 | P49448 | Glutamate dehydrogenase 2, mitochondrial | 0.59 | 0.015 | -0.31 | 0.110 | -0.24 | 0.022 | 0.52 | 0.031 |
| FBP1 | P09467 | Fructose-1,6-bisphosphatase 1 | 0.59 | 1.34E-11 | -0.19 | 0.036 | 0.15 | 6.74E-03 | 0.25 | 7.52E-04 |
| TRPS1 | Q9UHF7 | Zinc finger transcription factor Trps1 | 0.59 | 0.019 | -1.46 | 1.64E-04 | -1.93 | 4.54E-09 | 1.06 | 5.06E-04 |
| C7orf50 | Q9BRJ6 | Uncharacterized protein C7orf50 | 0.59 | 2.58E-06 | -0.60 | 1.25E-04 | -0.69 | 1.78E-08 | 0.68 | 4.23E-06 |
| EDRF1 | Q3B7T1 | Erythroid differentiation-related factor 1 | 0.59 | 0.049 | 0.35 | 0.030 | 0.31 | 0.091 | 0.63 | 3.92E-03 |
| CISD1 | Q9NZ45 | CDGSH iron-sulfur domain-containing protein 1 | 0.58 | 7.58E-03 | -0.44 | 3.39E-03 | -0.80 | 6.50E-03 | 0.94 | 0.013 |
| TBC1D15 | Q8TC07 | TBC1 domain family member 15 | 0.58 | 5.52E-06 | 0.08 | 0.095 | 0.23 | 1.01E-03 | 0.43 | 5.75E-03 |
| MAPT | P10636 | Microtubule-associated protein tau | 0.58 | 0.011 | -0.28 | 0.020 | 0.03 | 0.103 | 0.27 | 8.94E-03 |


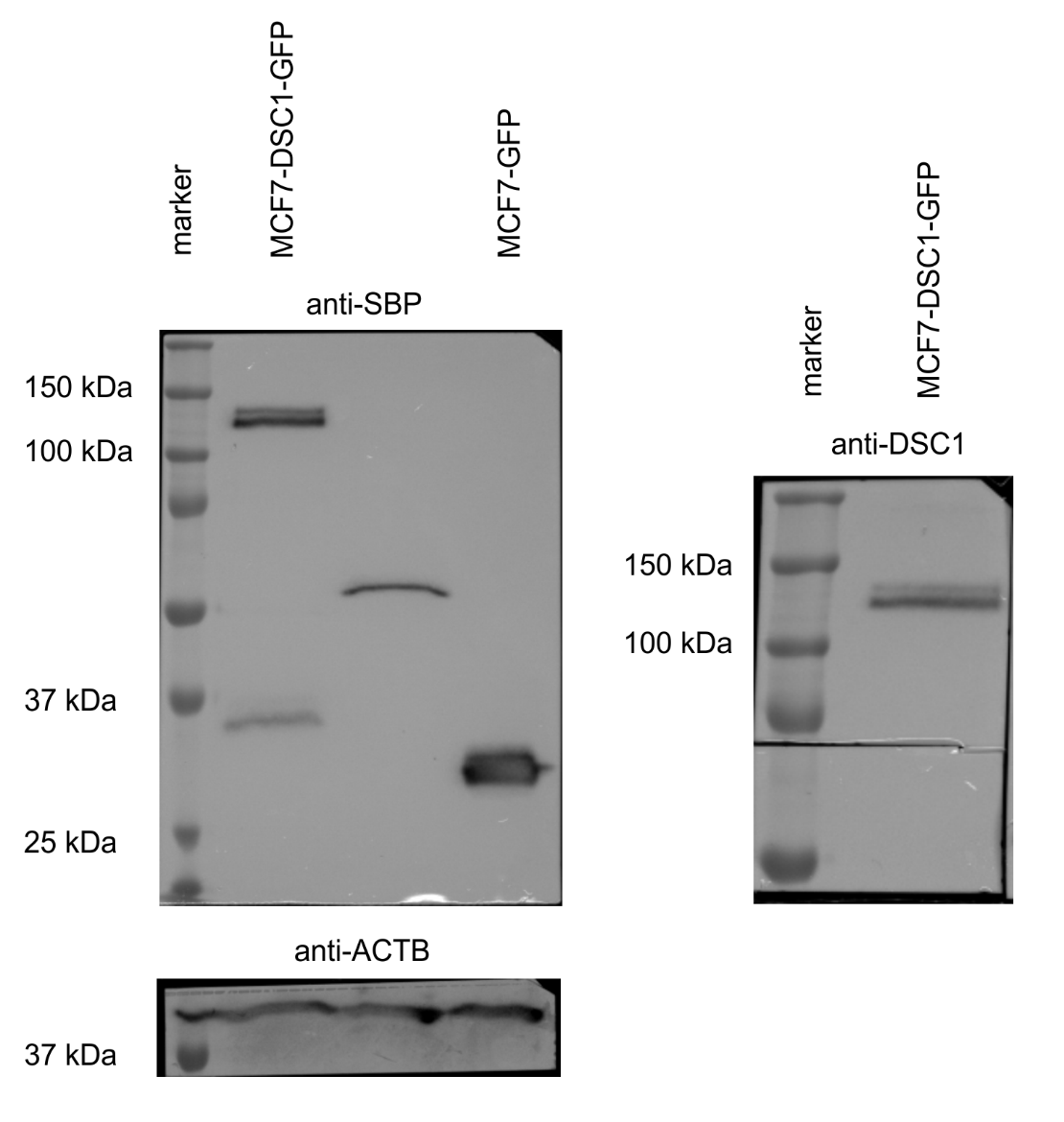


**Fig. S3** Western blot verification of SBP tag presence in control MCF7-GFP cells and MCF7-DSC1-GFP cells used for pull-down identification of DSC1 protein interaction partners. Antibodies: Anti-SBP-tag (left), Anti-DSC1 (right) and Anti-ACTB (down).
